# Supplementary material for: Targeting glutamine-addiction and overcoming CDK4/6 inhibitor resistance in human esophageal squamous cell carcinoma
Source: Nat Commun. 2019 Mar 21;10:1296. doi: 10.1038/s41467-019-09179-w (PMC6428878; doi:10.1038/s41467-019-09179-w)
Supplement: Supplementary file 2 — Supplementary Information [file 41467_2019_9179_MOESM2_ESM.pdf]

Supplementary Information

Targeting Glutamine-addiction and Overcoming CDK4/6 Inhibitor Resistance in Human Esophageal Squamous Cell Carcinoma

Qie et al.

Supplementary Figures

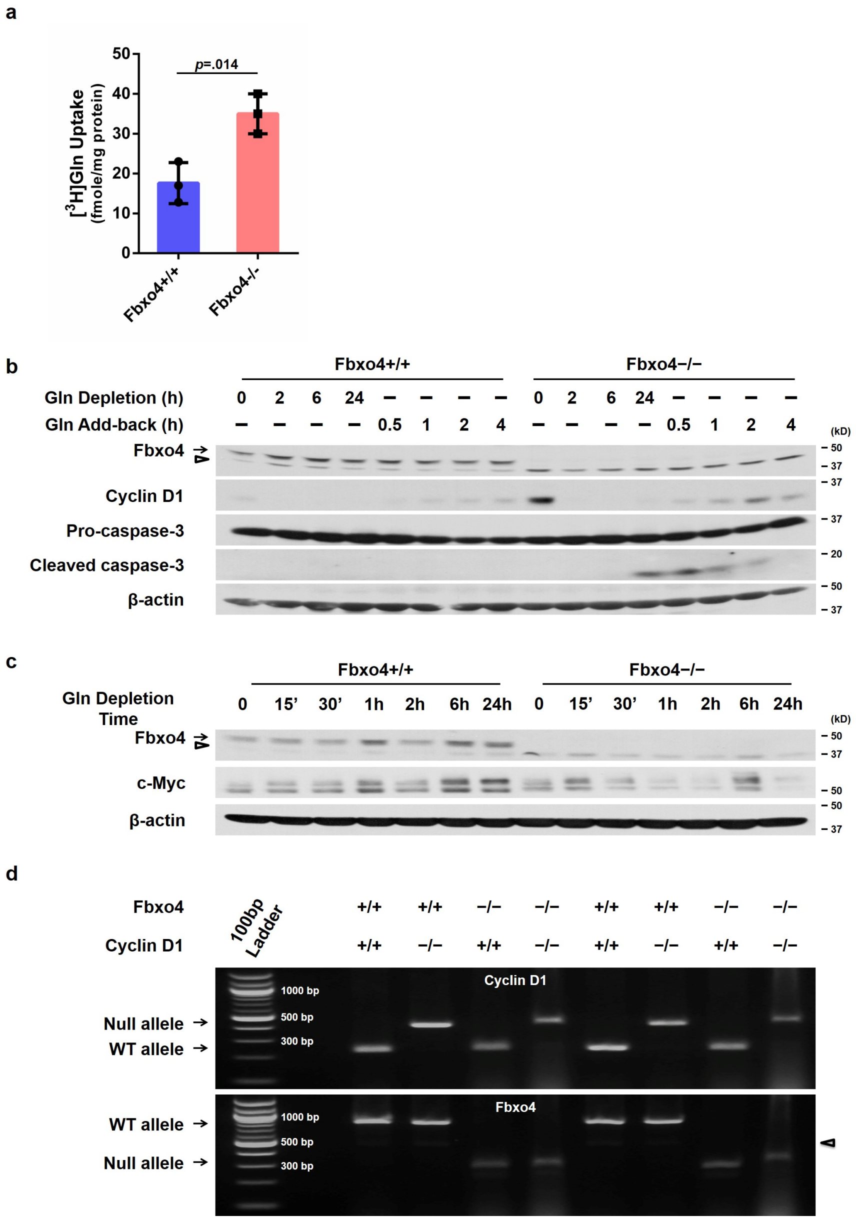

Supplementary Figure 1. Dysregulated Fbxo4-cyclin D1 enhances the sensitivity to Gln-depletion. (a) Reduced [^{3}H]Gln uptake in *Fbxo4*+/+ relative to −/− MEFs (1×10^5 cells used). Data represent as mean±s.d., two-tailed Student *t* test was used to compare means (*n*=3); *p* values are listed. (b) Western blot analysis shows less cleaved caspase-3 in *Fbxo4*+/+ than that in −/− MEFs upon Gln-depletion/re-supplementation. (c) Western blot analysis demonstrates higher c-Myc levels in *Fbxo4*+/+ than that in −/− MEFs upon Gln-depletion. (d) Genotyping of different MEFs. Two pairs of MEFs with different genetic backgrounds are shown. For cyclin D1, ~224bp band means WT, ~391bp band means Null; For Fbxo4, ~792bp band means WT, ~300bp band means Null. Arrow: interested band; Open triangle: non-specific band.

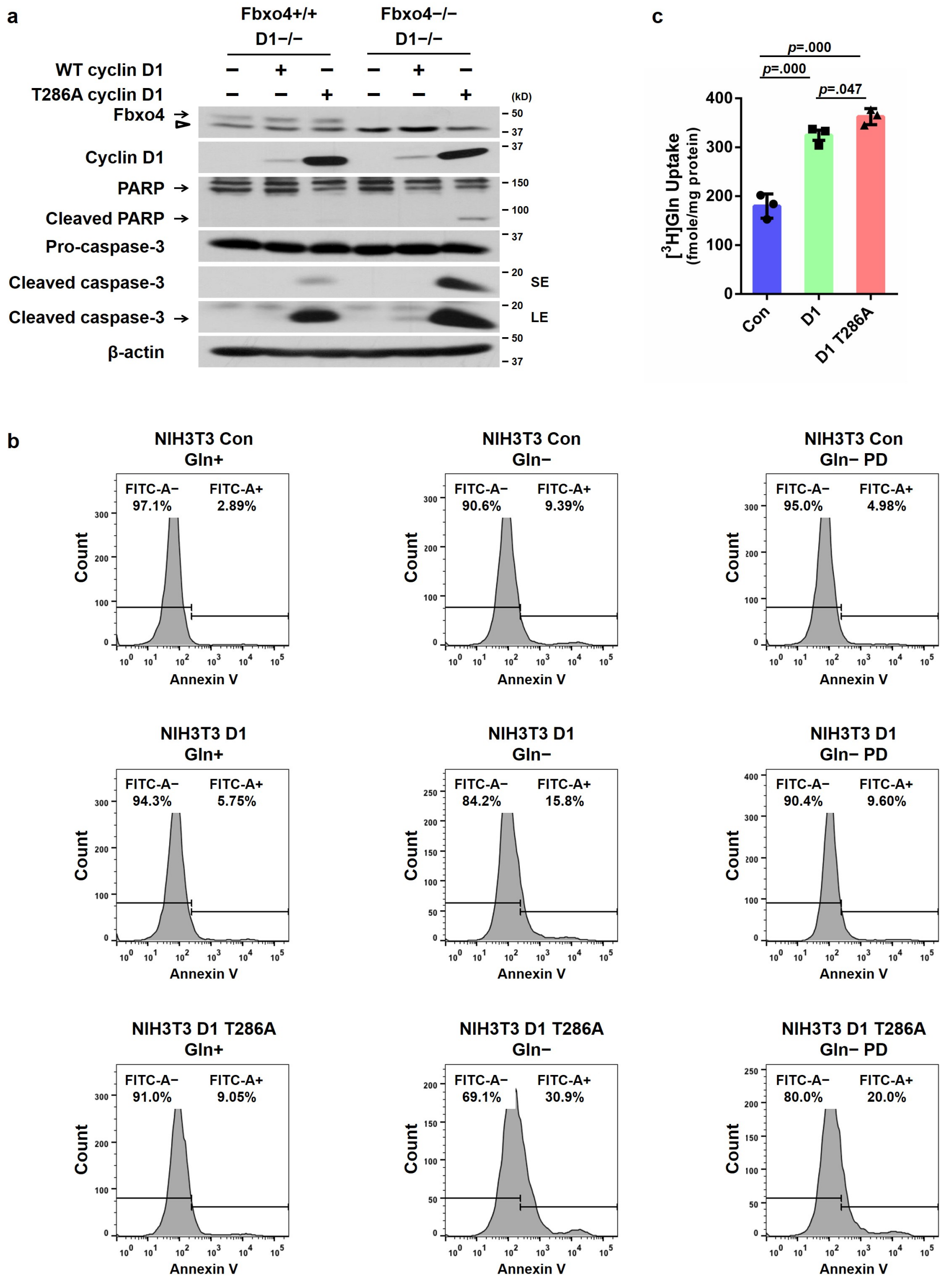

Supplementary Figure 2. Cyclin D1 is a downstream factor of Fbxo4 that regulates Gln-addiction. (a) Ectopic cyclin D1 expression promotes the cleavage of PARP and caspase-3 upon 24h Gln-depletion in MEFs. SE, short exposure; LE, long exposure. Arrow: interested band; Open triangle: non-specific band. (b) Palbociclib (PD) suppresses apoptosis of NIH3T3 cells with ectopic cyclin D1 expression upon indicated treatment for 48h. (c) Ectopic cyclin D1 enhances [^{3}H]Gln uptake in NIH3T3 cells (1×10^5 cells used). Data represent as mean±s.d., one-way ANOVA was used to compare means with Bonferroni as Post Hoc test (n=3); p values are listed.

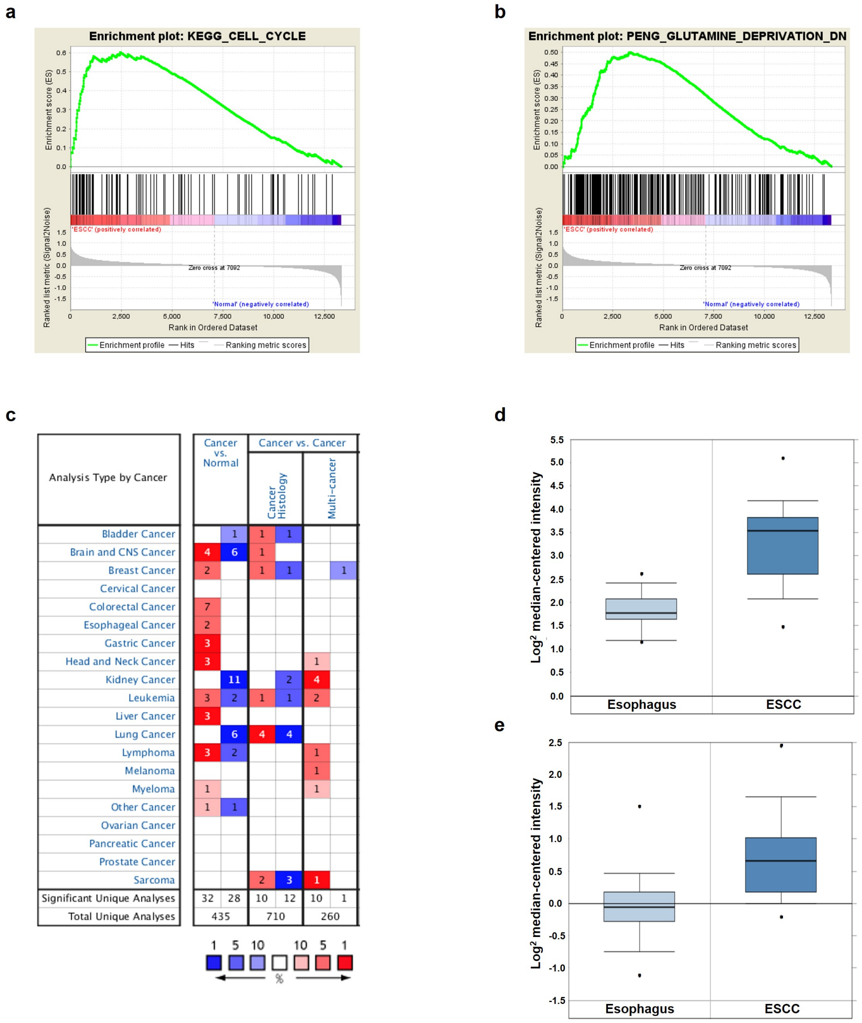

Supplementary Figure 3. GSEA and Oncomine analyses of gene expression in human ESCC compared to normal tissues. (a) GSEA enrichment plot shows the expression profiles of ESCC versus normal tissues of gene set “Cell Cycle Regulation” in NCBI GEO dataset (GSE20347): NES=1.4447355, FDR q-val=0.55833924. (b) GSEA enrichment plot shows the expression profiles of ESCC versus normal tissues of gene set related to “Gln Metabolism” in NCBI GEO dataset (GSE20347): NES=1.4435372, FDR q-val=0.12038835. (c) Oncomine analysis indicates the expression of *GLS1* across different tumour types when comparing cancer vs normal tissues (Access Date: 5/15/2018). (d) Comparison of *GLS1* expression between ESCC and normal tissues in Hu Esophagus. Fold change: 2.648, *t*-Test 5.902, *p* value: 3.12E-6. (e) Comparison of *GLS1* expression between ESCC and normal tissues in Su Esophagus 2. Fold change: 1.715, *t*-Test 6.726, *p* value: 6.40E-10. All data represent mean±s.d. and were analyzed by two-tailed Student *t* test. Oncomine Box-and-Whisker plots: median values are shown as horizontal bars; the upper and lower part of the box show the 75th percentile and the 25th percentile respectively; the upper and lower part of the bar show the 90th percentile and the 10th percentile respectively; the points show outlier values.

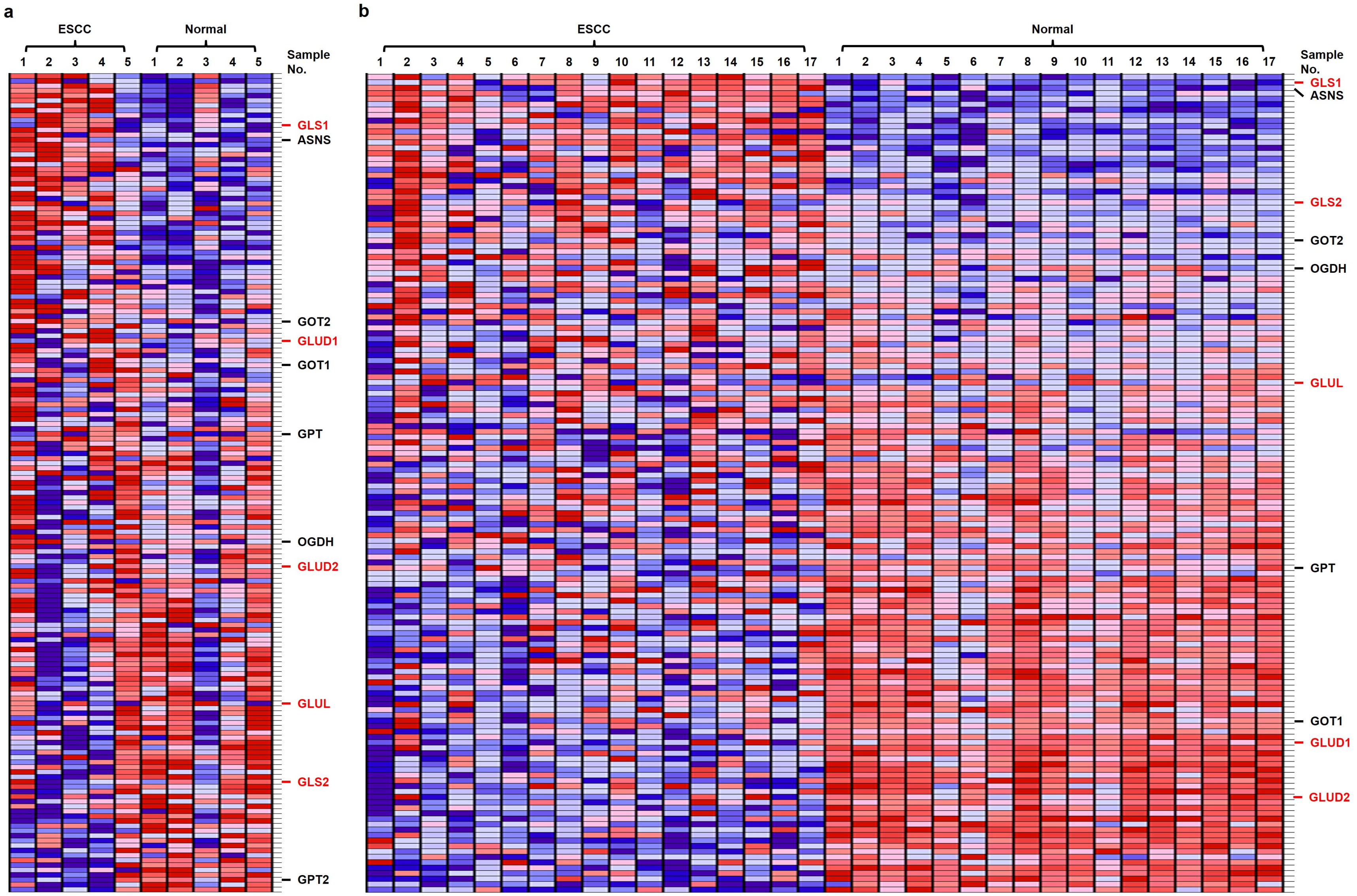

Supplementary Figure 4. Alteration of Gln metabolism genes in ESCC versus normal tissues. (a) Blue-Pink O' Gram in the Space of the Analyzed GeneSet with NCBI GEO (GSE100942); the comparison of Gln metabolism genes between ESCC and normal tissues. (b) Blue-Pink O' Gram in the Space of the Analyzed GeneSet with NCBI GEO (GSE20347); the comparison of Gln metabolism genes between ESCC and normal tissues. Red colour indicates gene upregulation; blue colour indicates gene downregulation.

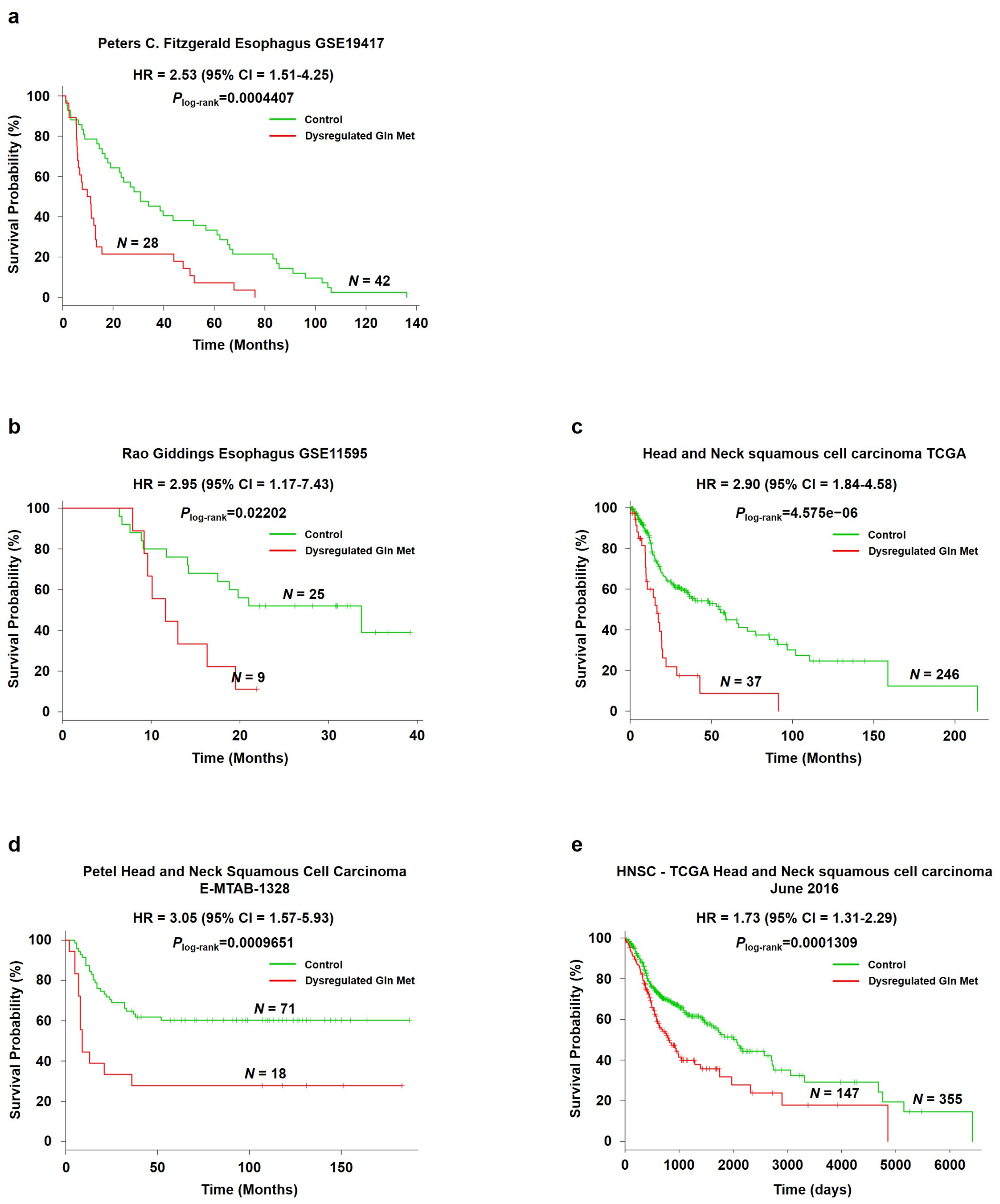

Supplementary Figure 5. Expression of Gln metabolism genes correlates with the survival of patients suffering from esophageal cancer or Head and Neck squamous cell carcinoma. Data are generated from the SurvExpression-survival database. (a) Peters C. Fitzgerald Esophagus GSE19417. (b) Rao Giddings Esophagus GSE11595. (c) Head and Neck squamous cell carcinoma TCGA. (d) Petel Head and Neck Squamous Cell Carcinoma E-MTAB-1328. (e) HNSC - TCGA Head and Neck squamous cell carcinoma June 2016. Kaplan-Meier survival curves were produced and the Log-Rank test was performed to compare the statistical significance. *N*, the numbers of patients in the listed group; Dysregulated Gln Met: dysregulated Gln metabolism; HR: Hazard Ratio; CI: Confidence Interval.

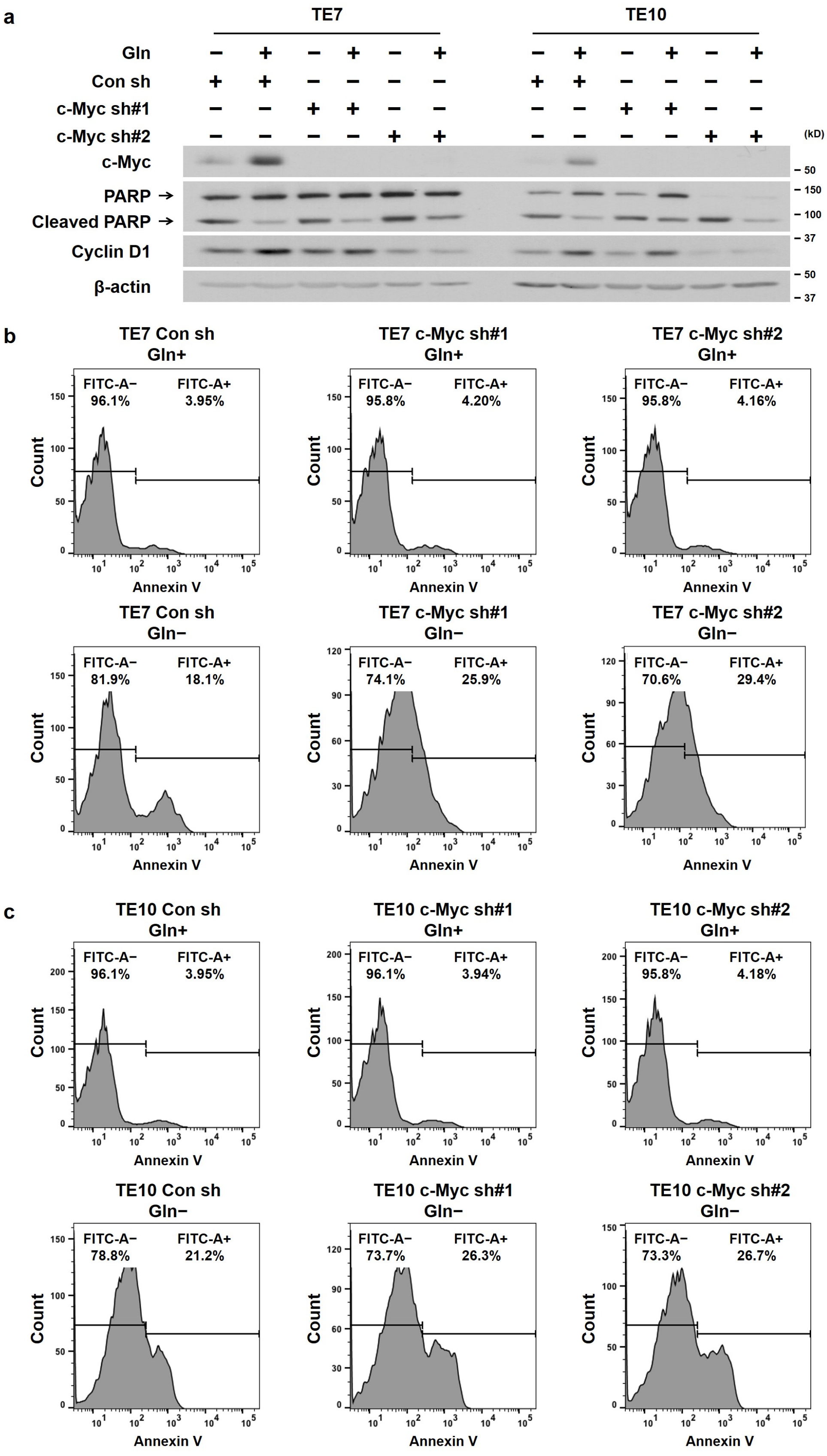
a
TE7
TE10
Gln
Con sh
c-Myc sh#1
c-Myc sh#2
(kD)
c-Myc
PARP
Cleaved PARP
Cyclin D1
β-actin
b
TE7 Con sh Gln+
FITC-A− 96.1% FITC-A+ 3.95%
TE7 c-Myc sh#1 Gln+
FITC-A− 95.8% FITC-A+ 4.20%
TE7 c-Myc sh#2 Gln+
FITC-A− 95.8% FITC-A+ 4.16%
TE7 Con sh Gln−
FITC-A− 81.9% FITC-A+ 18.1%
TE7 c-Myc sh#1 Gln−
FITC-A− 74.1% FITC-A+ 25.9%
TE7 c-Myc sh#2 Gln−
FITC-A− 70.6% FITC-A+ 29.4%
c
TE10 Con sh Gln+
FITC-A− 96.1% FITC-A+ 3.95%
TE10 c-Myc sh#1 Gln+
FITC-A− 96.1% FITC-A+ 3.94%
TE10 c-Myc sh#2 Gln+
FITC-A− 95.8% FITC-A+ 4.18%
TE10 Con sh Gln−
FITC-A− 78.8% FITC-A+ 21.2%
TE10 c-Myc sh#1 Gln−
FITC-A− 73.7% FITC-A+ 26.3%
TE10 c-Myc sh#2 Gln−
FITC-A− 73.3% FITC-A+ 26.7%
Count
Annexin V

Supplementary Figure 6. *c-Myc* knockdown does not suppress Gln-addiction in ESCC cells. (a) Western blot analysis reveals *c-Myc* knockdown does not reduce PARP cleavage in ESCC cells upon 24h Gln-depletion. Arrow: interested band. (b) FACS analysis reveals that *c-Myc* knockdown does not compromise apoptosis of TE7 cells upon 48h Gln-depletion. (c) FACS analysis demonstrates that *c-Myc* knockdown does not compromise apoptosis of TE10 cells upon 48h Gln-depletion.

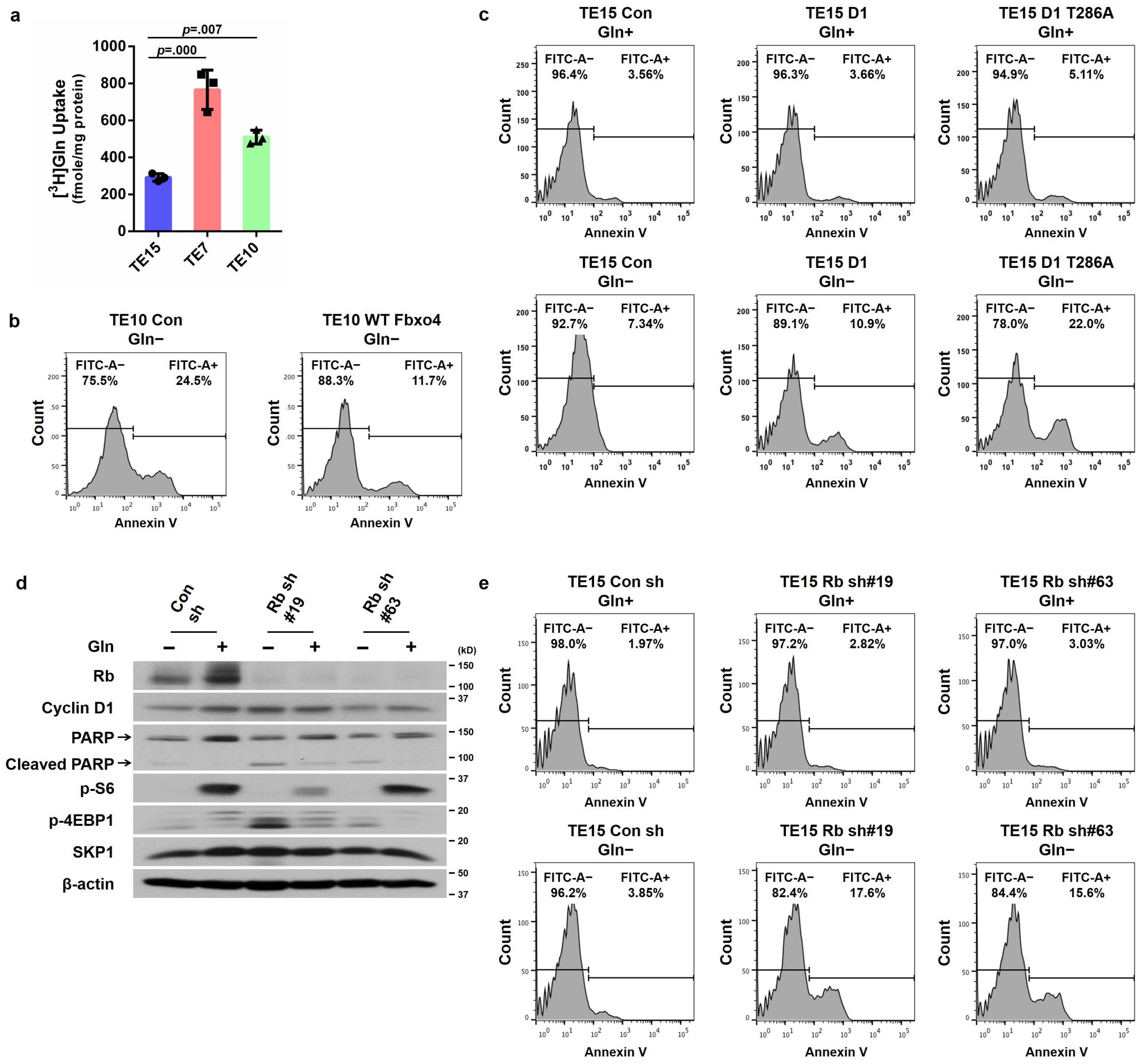

Supplementary Figure 7. Gln-depletion induces apoptosis in ESCC cells with dysregulated Fbxo4-cyclin D1. (a) [^{3}H]Gln uptake is increased in TE7 and TE10 cells relative to TE15 cells (2×10^5 cells used). Data represent as mean±s.d., one-way ANOVA was used to compare means with Bonferroni as Post Hoc test (n=3); p values are listed. (b) FACS analysis detects less apoptosis in TE10 cells with Fbxo4 expression upon 48h Gln-depletion. (c) FACS analysis demonstrates ectopic cyclin D1 promotes apoptosis of TE15 cells upon 48h Gln-depletion. (d) *Rb* knockdown induces PARP cleavage in TE15 cells upon 24h Gln-withdrawal. Arrow: interested band. (e) FACS analysis reveals *Rb* knockdown promotes cell apoptosis in TE15 cells upon 48h Gln-depletion.

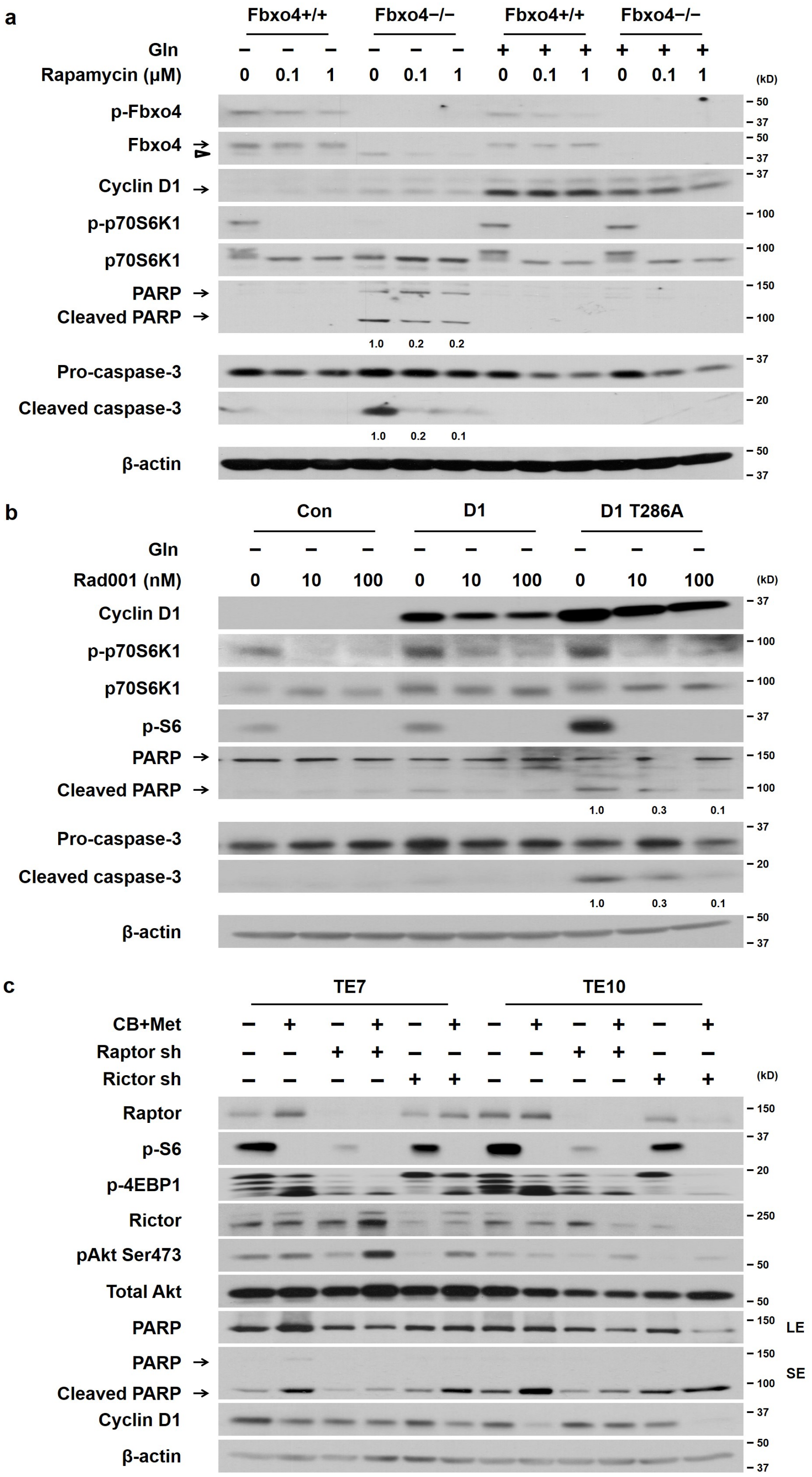

Supplementary Figure 8. mTORC1 inhibition reduces cell apoptosis. (a) Rapamycin suppresses mTORC1 activation and apoptosis in *Fbxo4−/−* MEFs. (b) Rad001 suppresses mTORC1 activity and apoptosis in NIH3T3 cells expressing ectopic cyclin D1. (c) *Raptor*

knockdown partially rescues apoptosis-induced by CB-839 plus metformin. SE, short exposure; LE, long exposure. Arrow: interested band; Open triangle: non-specific band. The numbers below the bands indicate the intensity quantification of cleaved PARP or cleaved caspase-3 in the relevant lanes.

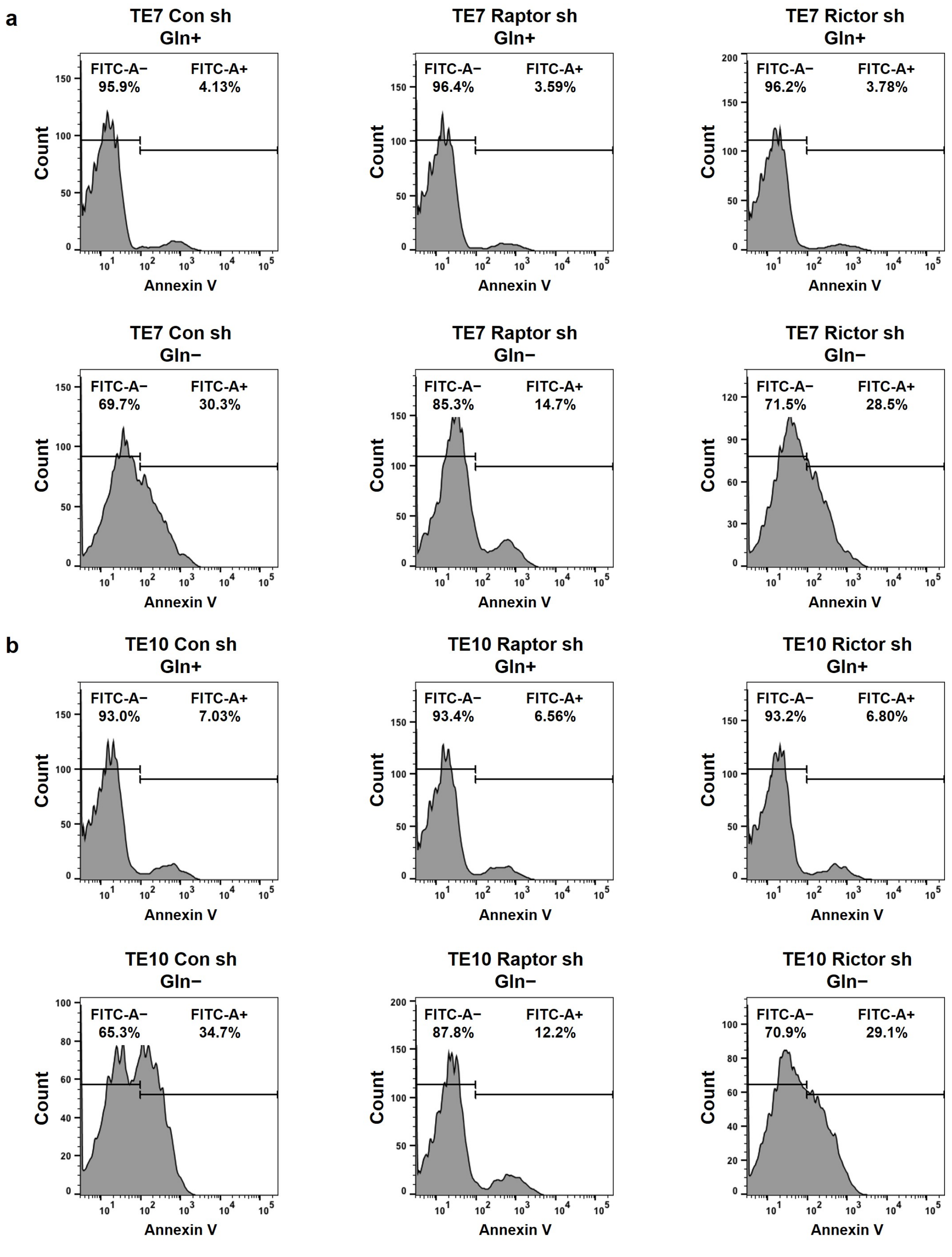

Supplementary Figure 9. *Raptor* knockdown suppresses cellular dependency on Gln in ESCC cells. (a) FACS analysis reveals reduced apoptosis in TE7 cells with *Raptor* knockdown upon 48h Gln-depletion. (b) FACS analysis reveals less apoptosis of TE10 cells with *Raptor* knockdown upon 48h Gln-depletion.

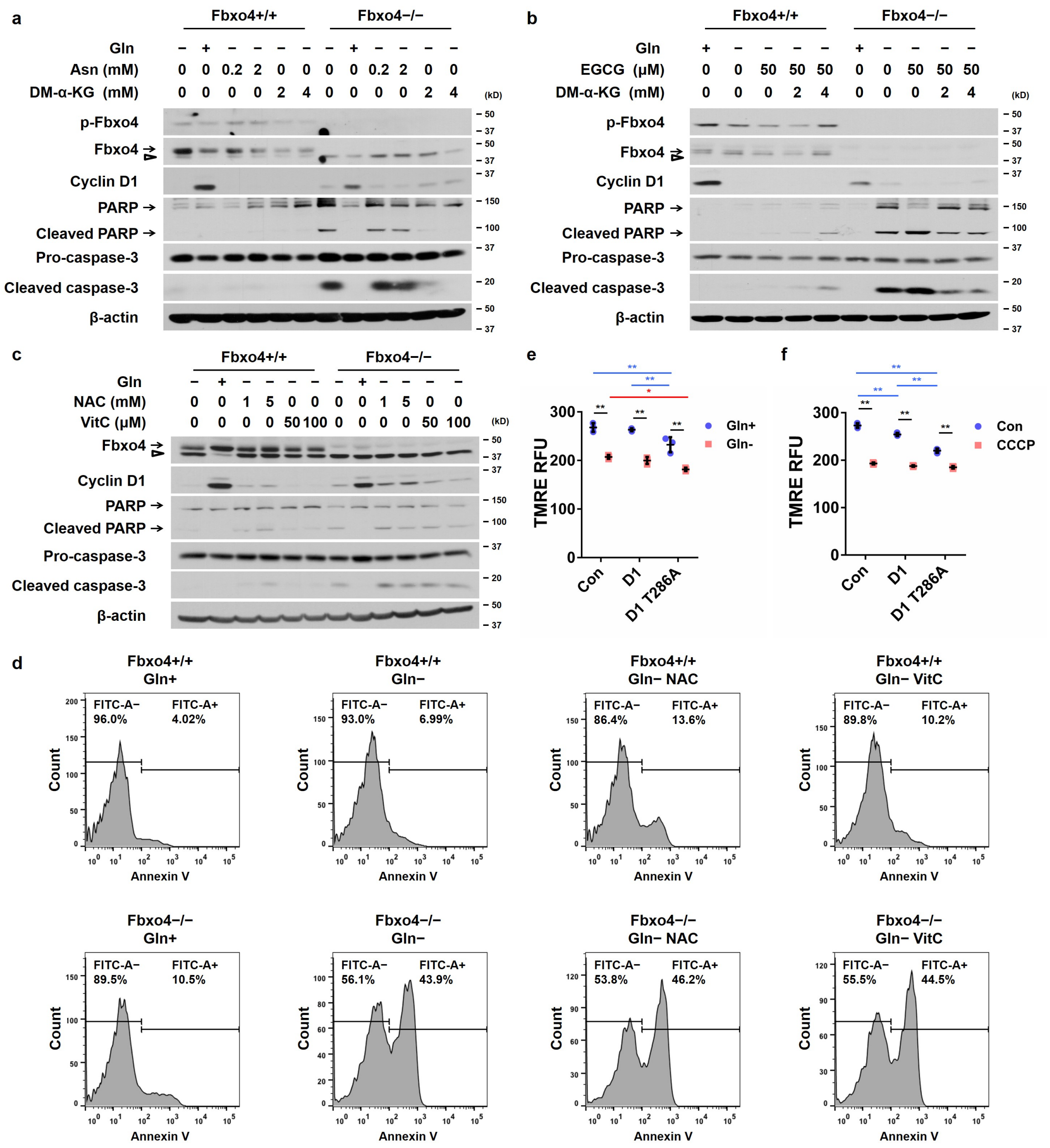

Supplementary Figure 10. Energetic metabolism in cells with dysregulated Fbxo4-cyclin D1. (a) Dimethyl-α-ketoglutarate (DM-α-KG) but not Asparagine (Asn) reduces apoptosis in *Fbxo4*−/− MEFs upon 24h Gln-depletion. (b) DM-α-KG reduces apoptosis-induced by EGCG, which inhibits the activity of glutamate dehydrogenase. (c) ROS scavengers, N-Acetyl-L-cysteine (NAC) and Vitamin C (VitC), have no anti-apoptotic effects in *Fbxo4*+/+ and −/− MEFs upon 24h Gln-depletion. (d) FACS analysis reveals that NAC and VitC cannot suppress apoptosis induced by 48h Gln-depletion. Concentration used: NAC, 5 mM; VitC, 100 μM. (e) Mitochondrial membrane potential is compromised in NIH3T3 cells with ectopic cyclin D1. (f) CCCP treatment as positive control in NIH3T3 cells. All data represent as mean±s.d., one-way ANOVA was used to compare means with Bonferroni as Post Hoc test (n=3). *, p<0.05; **, p<0.01. Arrow: interested band; Open triangle: non-specific band.

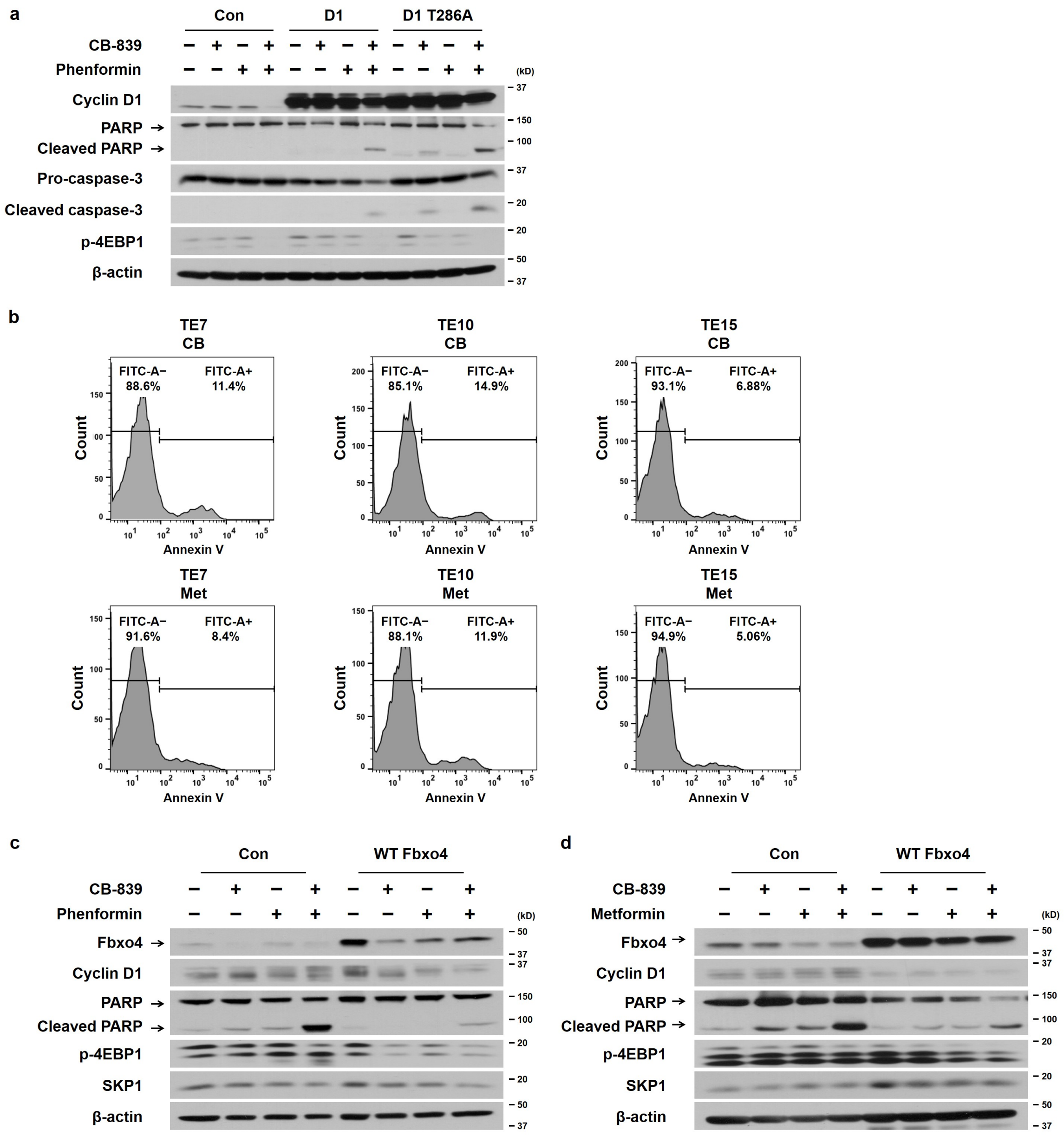

Supplementary Figure 11. Combined treatment induces cell apoptosis. (a) CB-839 and phenformin treatment induce more apoptosis in NIH3T3 cells with ectopic cyclin D1 expression. (b) FACS analysis reveals apoptosis in TE7, TE10 and TE15 cells upon 48h treatment with CB-839 or metformin (related to Figure 5e). (c & d) WT Fbxo4 suppresses cell apoptosis-induced by combined treatment in TE10 cells: CB-839+phenformin (c) and CB-839+metformin (d). Arrow: interested band.

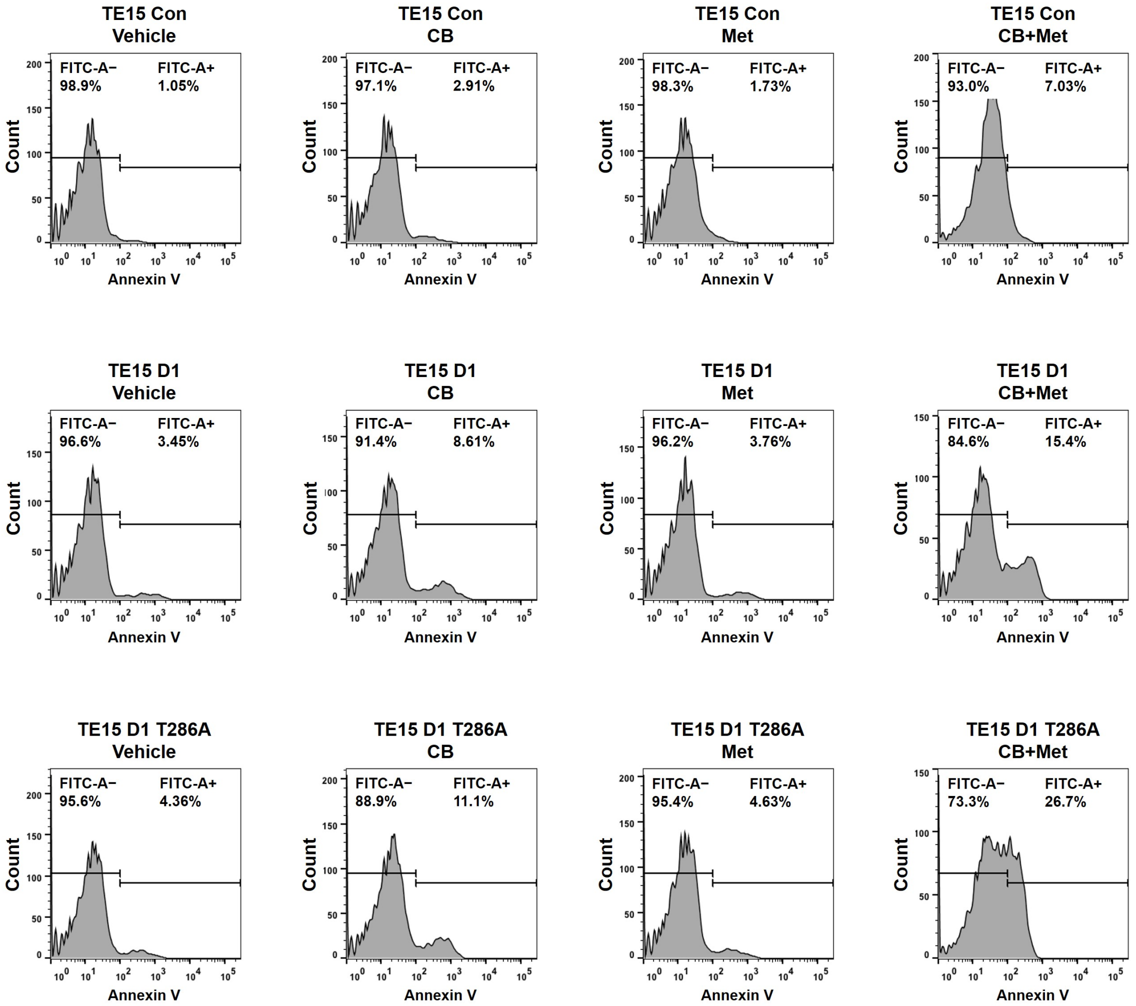

Supplementary Figure 12. FACS analysis reveals apoptosis upon relevant treatment. Increased apoptosis in TE15 cells with ectopic D1 and D1 T286A upon 48h treatment.

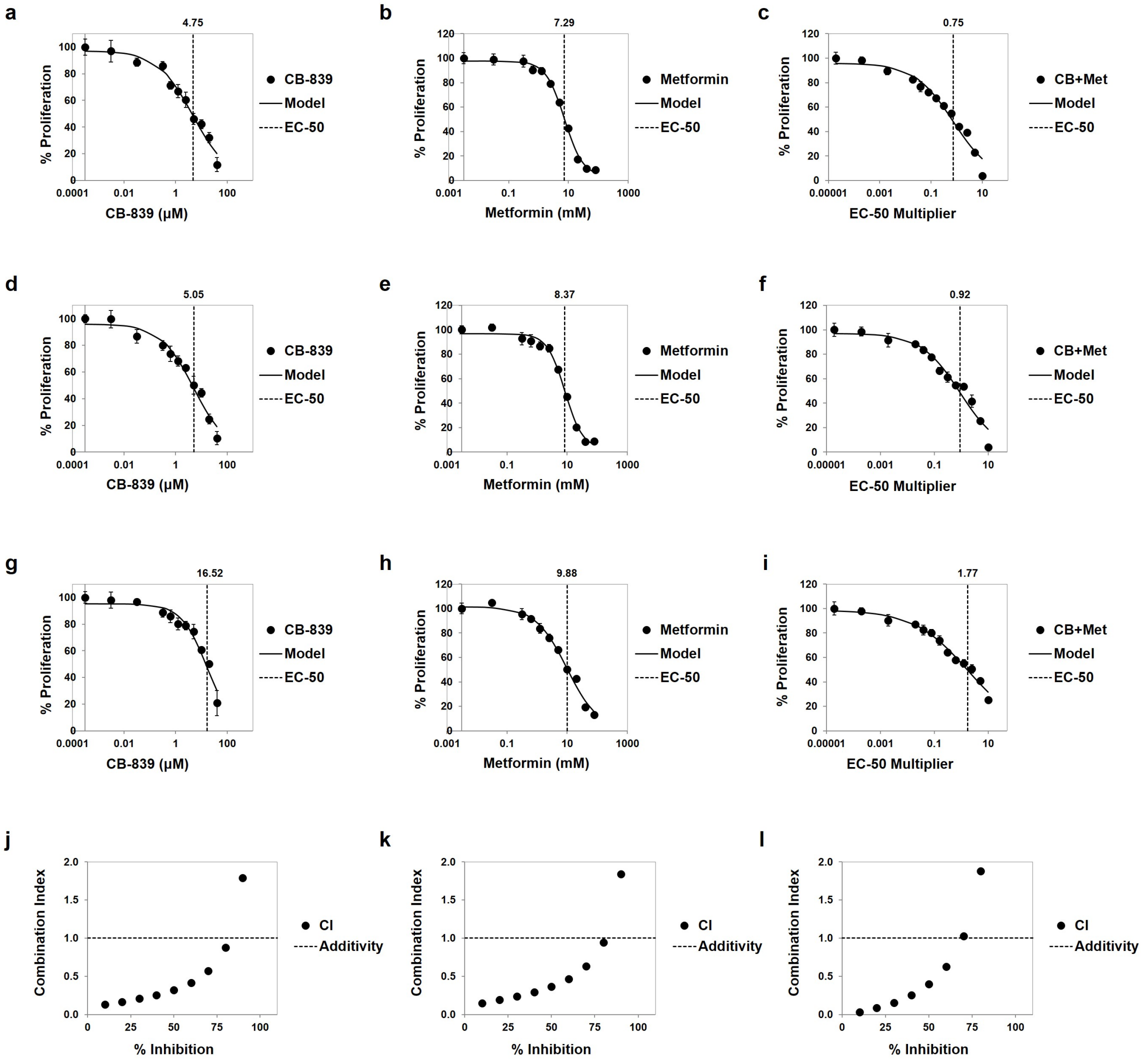

Supplementary Figure 13. Synergistic effects of CB-839 and metformin in ESCC cells. (a-i) EC50s of CB-839, metformin or their combination in TE7 (a-c), TE10 (d-f) and TE15 cells (g-i). (j-l) Demonstration of synergistic combination index (CI) of CB-839 and metformin in TE7 (j), TE10 (k) and TE15 (l) cells.

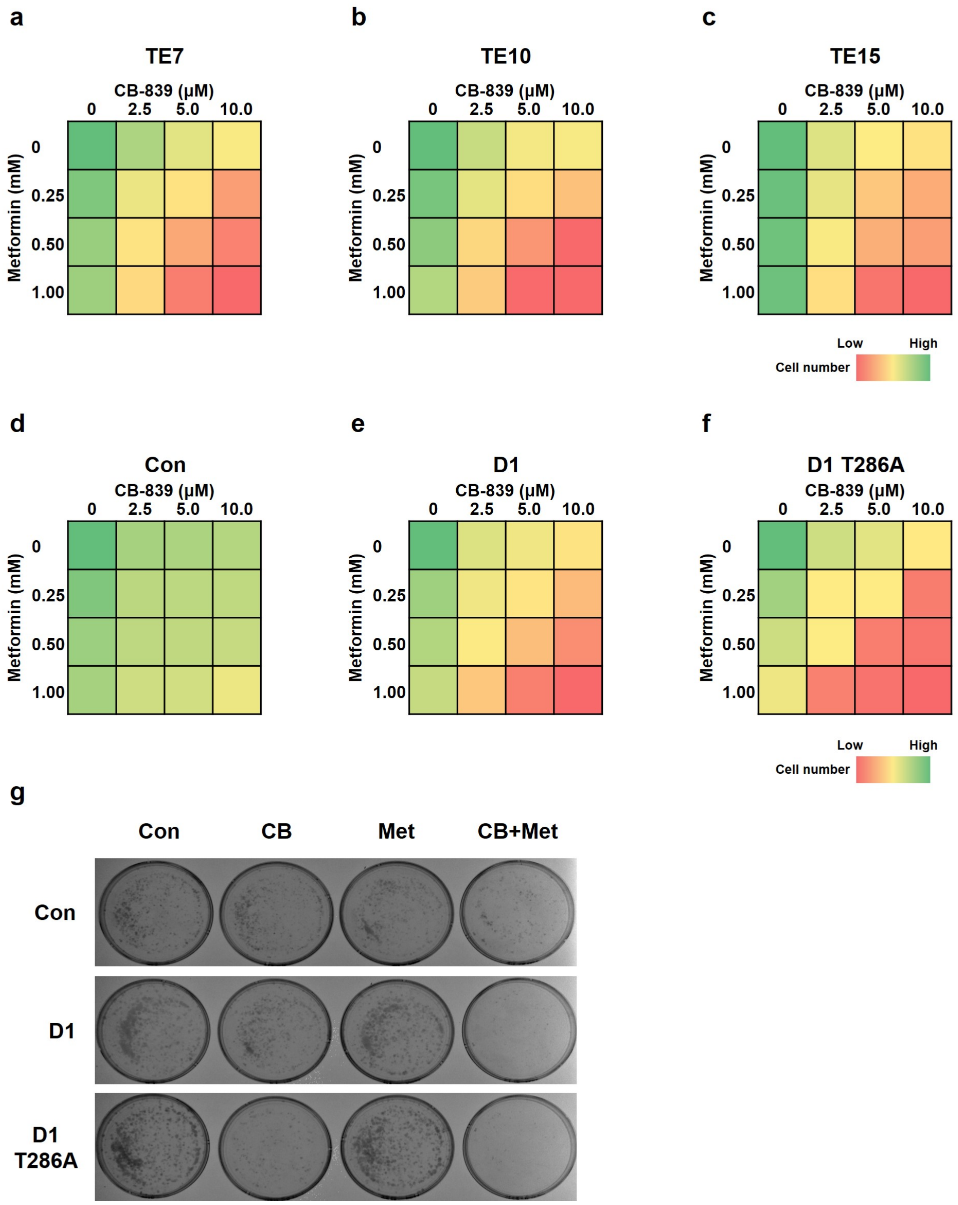

Supplementary Figure 14. Synergistic effects of combination treatment. (a-c) The suppressive effects of different combinations of CB-839 and metformin on TE7 (a), TE10 (b) and TE15 (c) cells. Cell proliferation was determined using CyQUANT® NF Cell Proliferation Assay Kit. Green colour equates with high cell number while red colour indicates low cell number. (d-f) The suppressive effects of different combinations of CB-839 and metformin on NIH3T3 cells with Control (d), D1 (e) and D1 T286A (f). Cell proliferation was measured using CyQUANT® NF Cell Proliferation Assay Kit. Green colour means high cell number while red colour indicates low cell number. (g) Clonogenic formation is suppressed in NIH3T3 cells by indicated treatment.

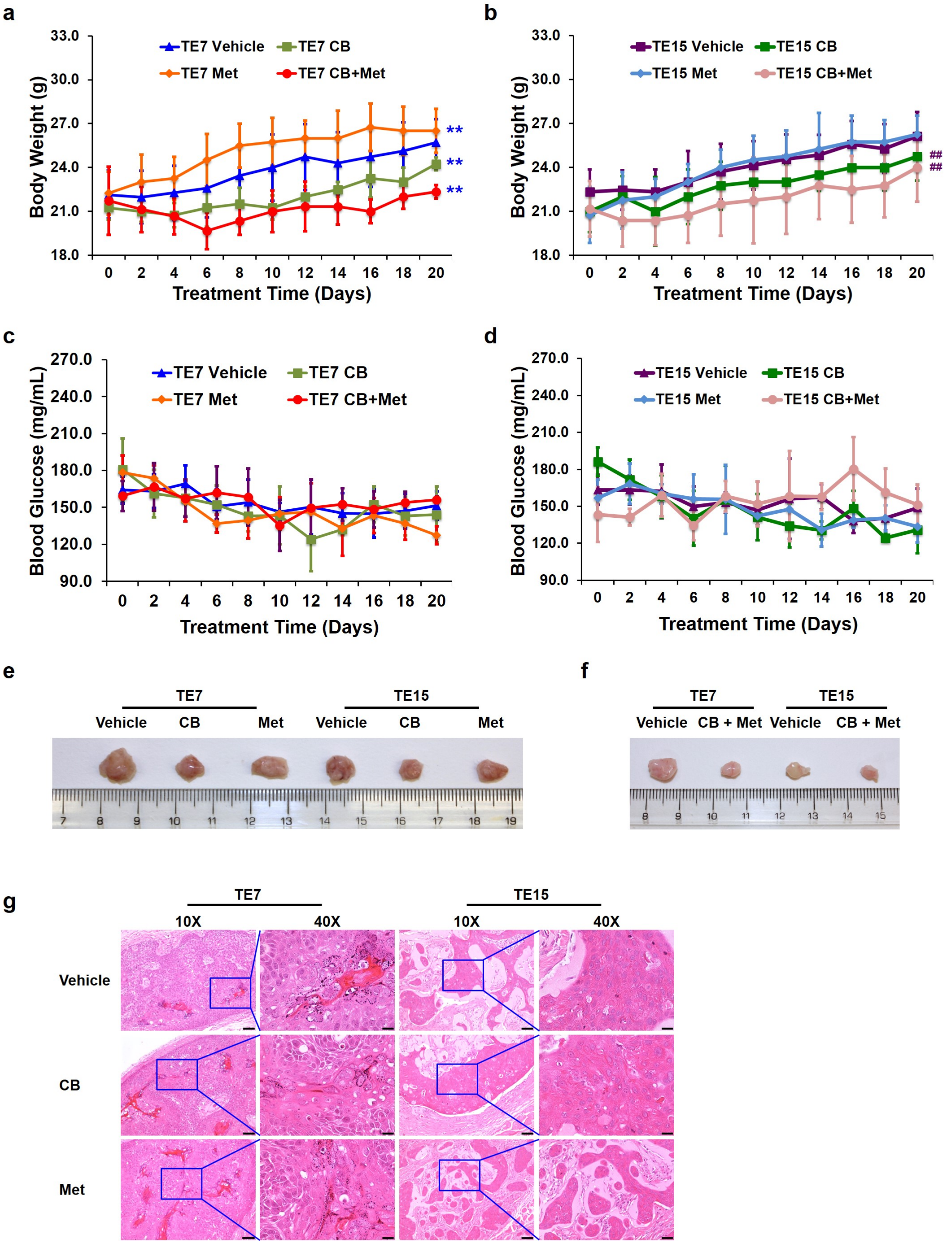

Supplementary Figure 15. The characteristics of TE7 and TE15 xenograft model. (a & b) Body weight curve after indicated treatment of TE7 (a) and TE15 (b) mice. Data represent as mean±s.d., two-way ANOVA was used to compare means with Bonferroni as Post Hoc test (*n*=4). **, *p*<0.01 (comparison between TE7 mice); ##, *p*<0.01 (comparison between TE15 mice). (c & d) Blood glucose levels in mice with indicated treatment of TE7 (c) and TE15 (d) mice. (e) Representative tumour xenografts from vehicle, CB-839 or metformin treated groups. (f) Representative tumour xenografts from vehicle and combined CB-839 & metformin treated groups. (g) H&E staining sections from tumour xenografts with indicated treatment. Scale bar, 10 μm.

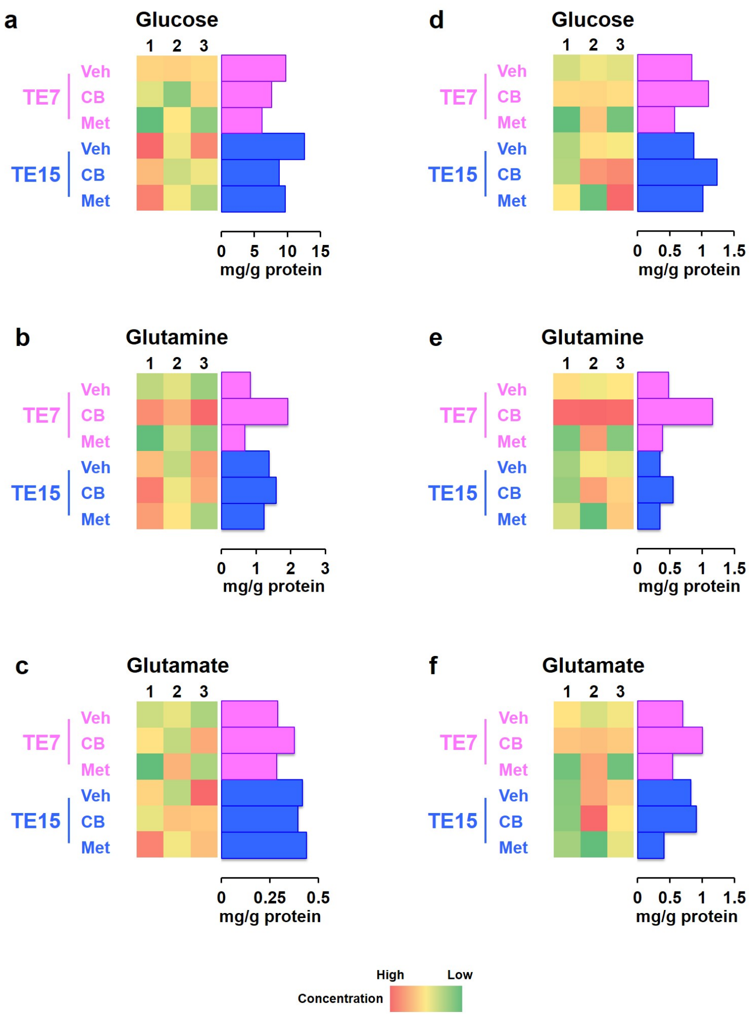

Supplementary Figure 16. NMR analysis of Glucose (Glc), Gln and Glutamate (Glu) levels in the livers and in the tumour xenografts. (a-c) Glc, Gln and Glu concentrations in liver tissues. (d-f) Glc, Gln and Glu concentrations in tumour xenografts. Green colour means low concentration while red colour indicates high concentration.

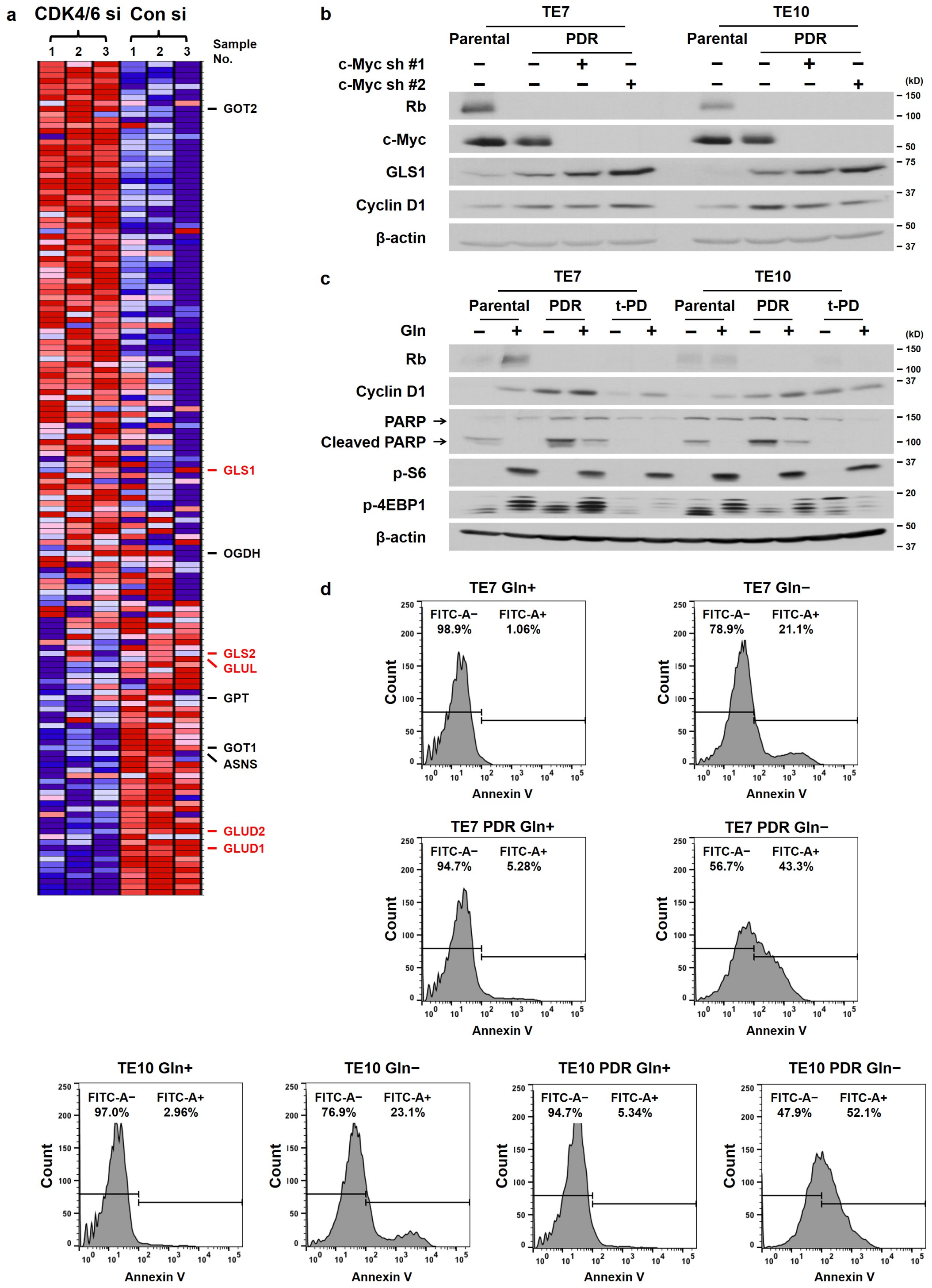

Supplementary Figure 17. Combined treatment overcomes palbociclib resistance in ESCC cells. (a) Alteration of Gln metabolism genes upon *CDK4/6* knockdown. The GEO datasets were downloaded from NCBI, normalized by R-Project Bioconductor, and analyzed by GSEA. Blue-Pink O' Gram in the Space of the Analyzed GeneSet with NCBI GEO (GSE84597), the comparison of Gln metabolism genes between *CDK4/6* siRNA and control siRNA transfected human HCT116 cells. Red colour indicates gene upregulation; blue colour indicates gene downregulation. (b) Western blot indicates *c-Myc* knockdown doesn't reduce GLS1 expression

in TE7PDR and TE10PDR cells. (c) Western blot demonstrates both TE7PDR and TE10PDR cells are more sensitive to Gln-depletion. t-PD, transiently treated with palbociclib for 4 days. Arrow: interested band. (d) FACS analysis reveals more apoptosis in PDR than in parental ESCC cells upon 48h Gln-depletion.

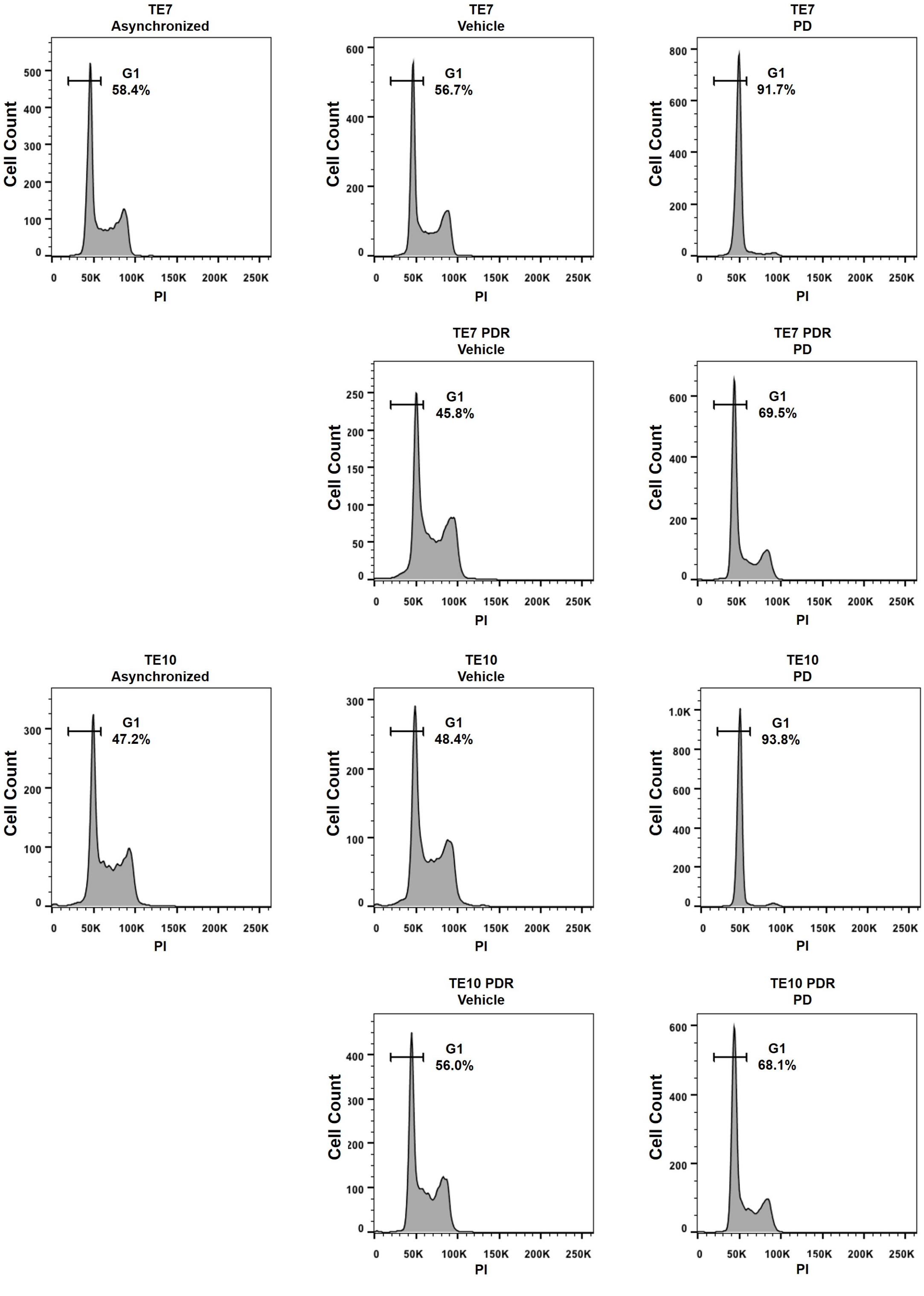

Supplementary Figure 18. The characterization of parental and PDR ESCC cells. Listed cells are untreated, or treated with vehicle or 1 μM palbociclib for 24h before Propidium Iodide (PI) staining and FACS analysis.

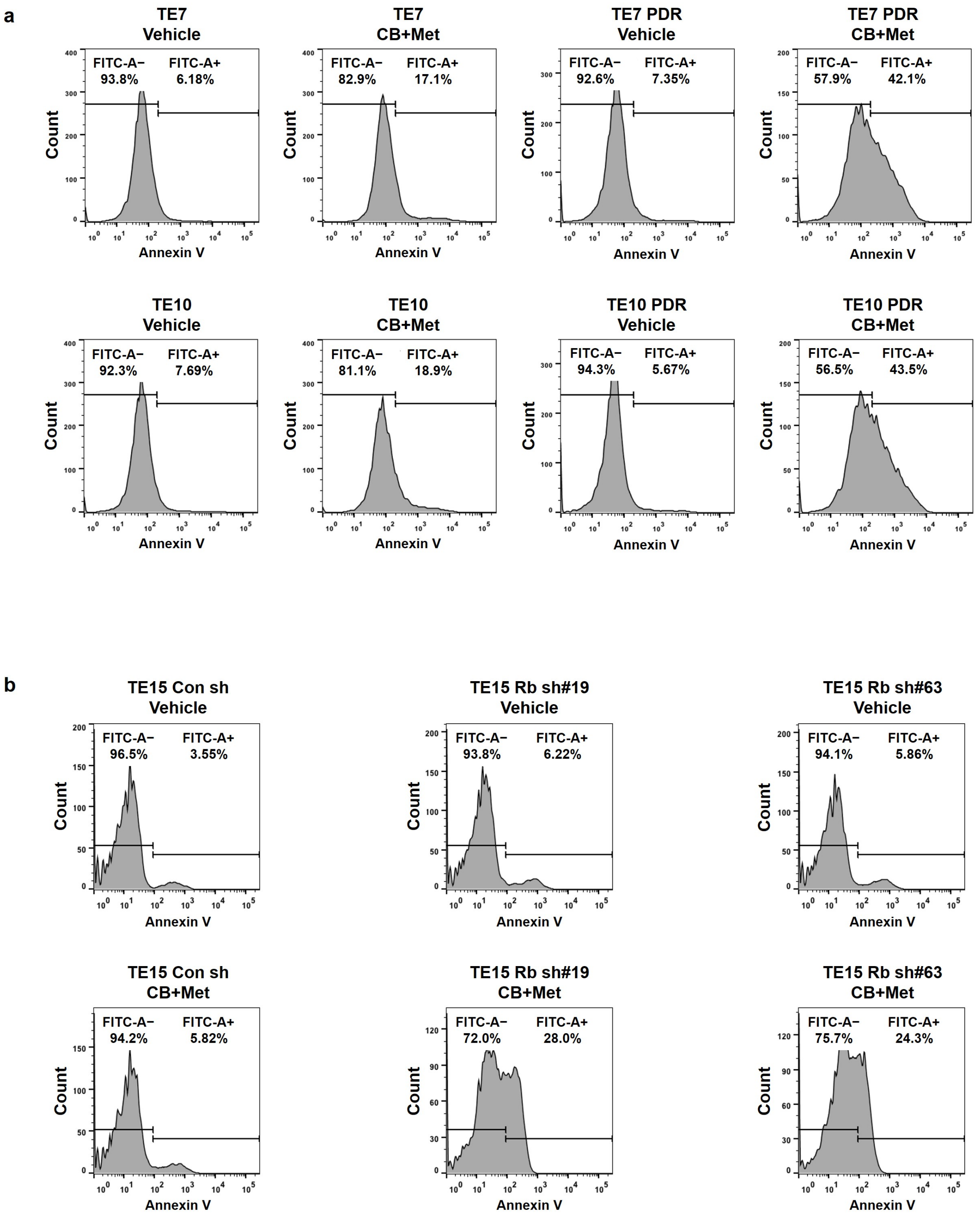

Supplementary Figure 19. FACS detection of apoptosis in ESCC cells. (a) FACS analysis reveals more apoptosis in PDR than in parental ESCC cells upon 48h combined treatment. (b) FACS reveals *Rb* knockdown promotes apoptosis in TE15 cells upon 48h combined treatment.

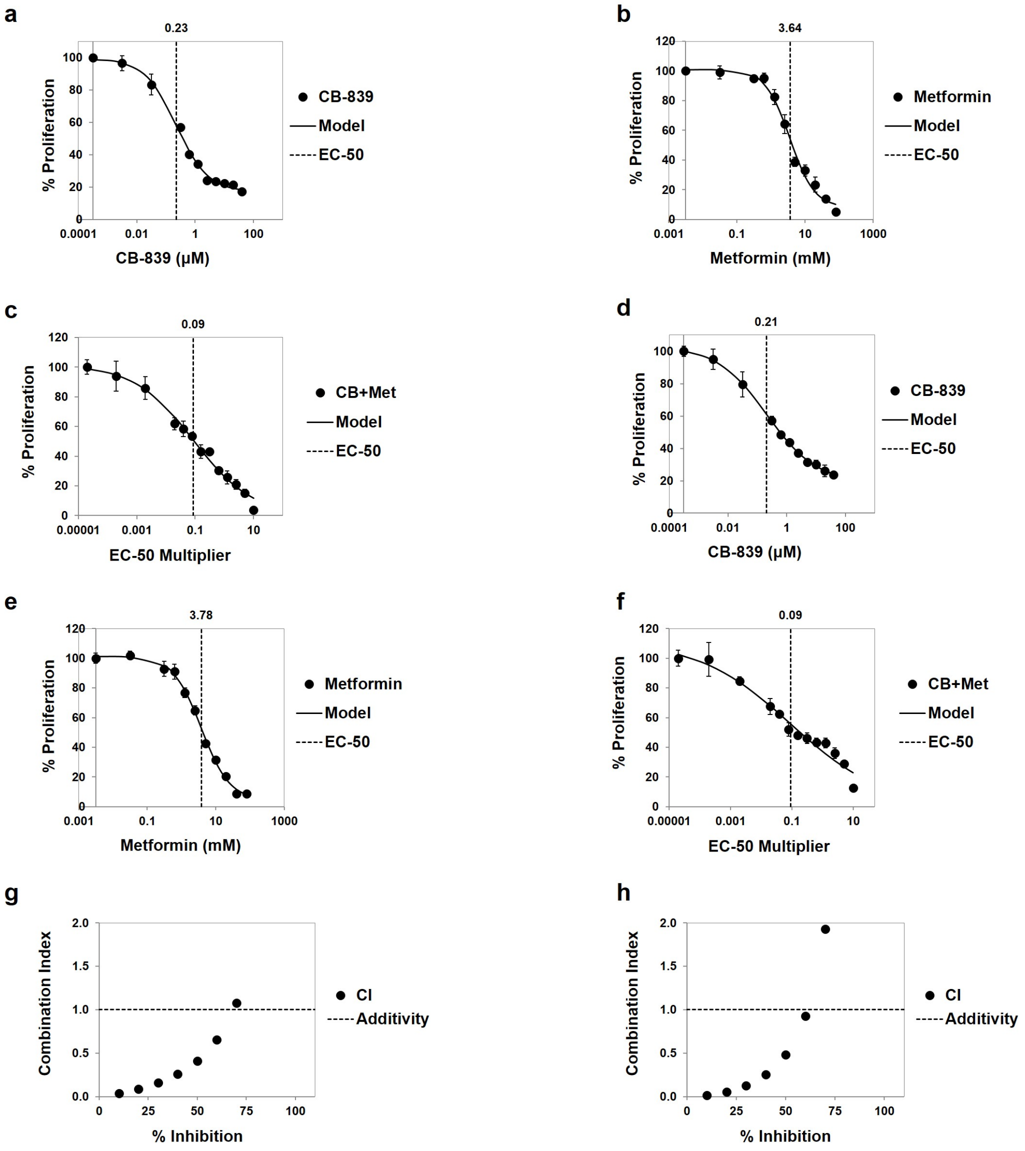

Supplementary Figure 20. The determination of EC50s in PDR cells. (a-f) EC50s of CB-839, metformin or their combination in TE7PDR cells (a-c) and TE10PDR cells (d-f). (g & h) Demonstration of synergistic CI of CB-839 and metformin in TE7PDR cells (g) and TE10PDR cells (h).

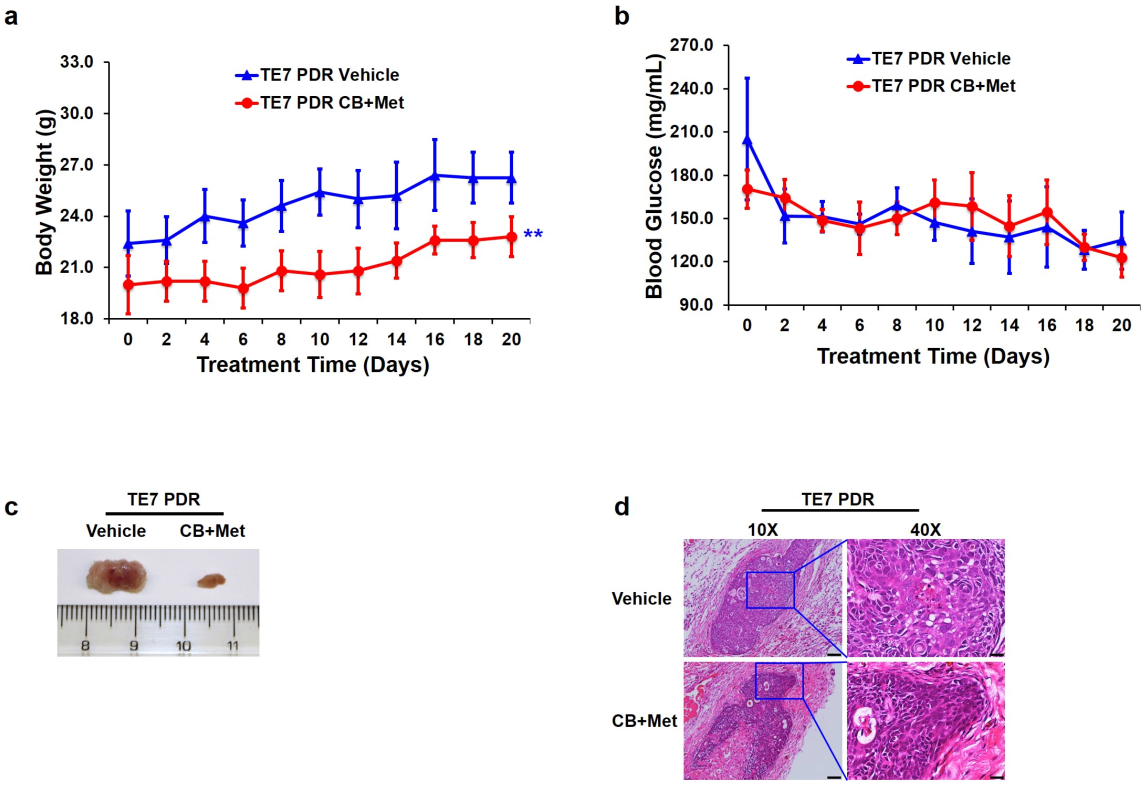

Supplementary Figure 21. The characteristics of TE7PDR xenograft model. (a) Body weight curve after indicated treatment. Data represent as mean±s.d., two-way ANOVA was used to compare means with Bonferroni as Post Hoc test (n=4). **, p<0.01. (b) Blood glucose levels with indicated treatment. (c) Representative tumour xenografts from vehicle and combined CB-839 & metformin treated groups. (d) H&E staining of sections from tumour xenografts with indicated treatment. Scale bar, 10 μm.

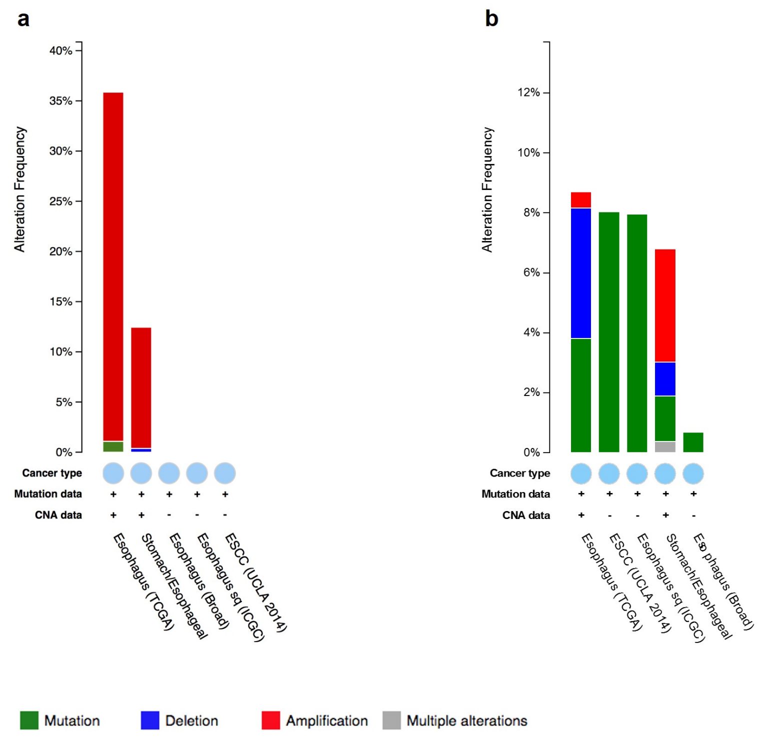

Supplementary Figure 22. cBioportal TCGA analysis of *CCND1* and *Rb1* gene status in human esophageal carcinoma samples. (a) *CCND1* gene status. (b) *Rb1* gene status.

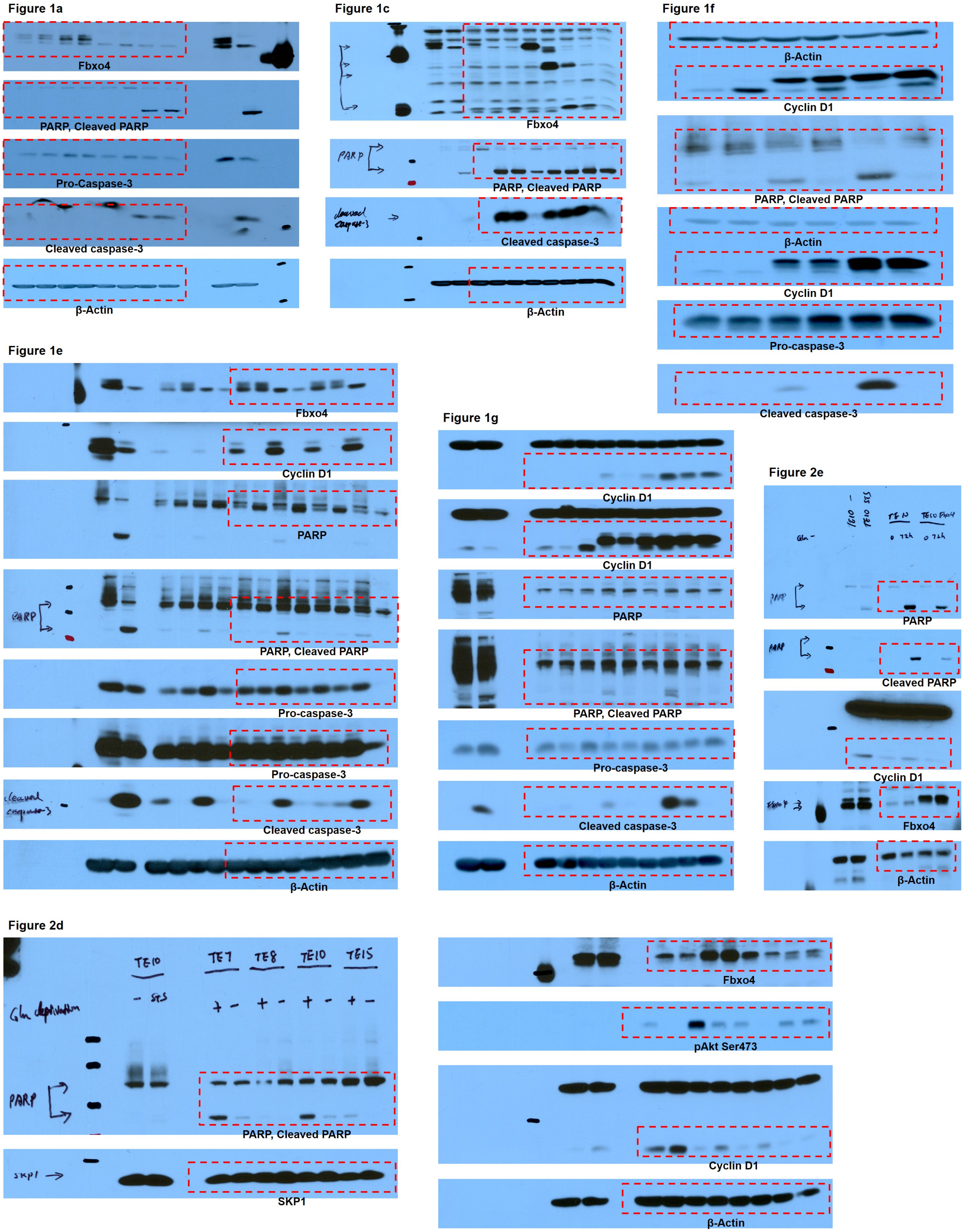

Supplementary Figure 23. Uncropped scans of the most important blots.

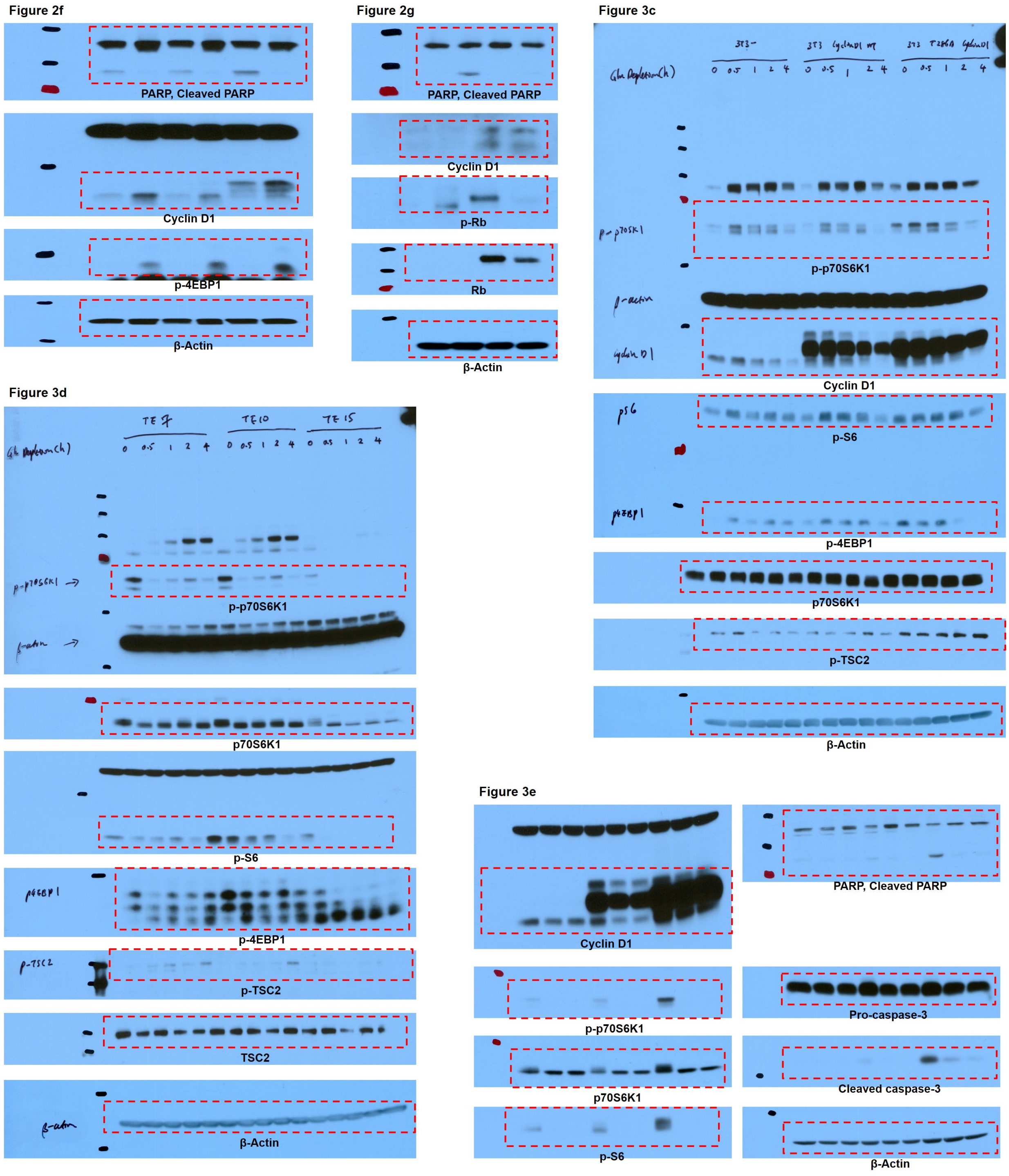

Supplementary Figure 23. Uncropped scans of the most important blots.

Figure 3f

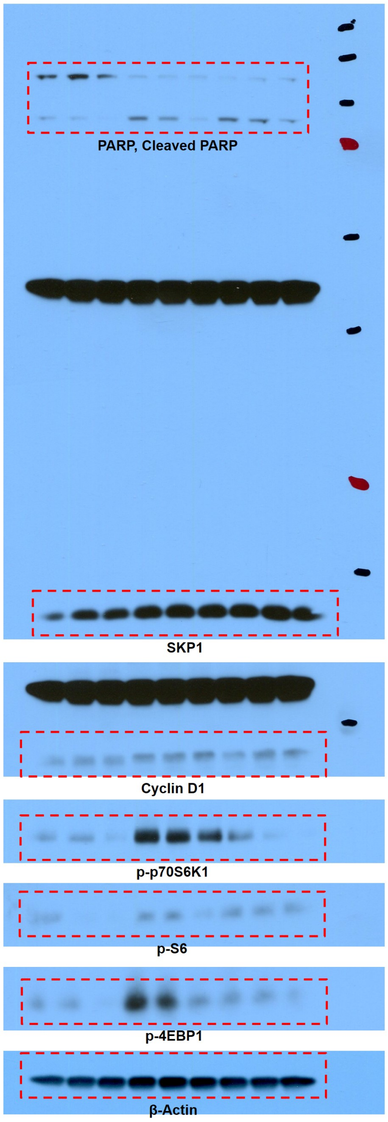

Figure 3g

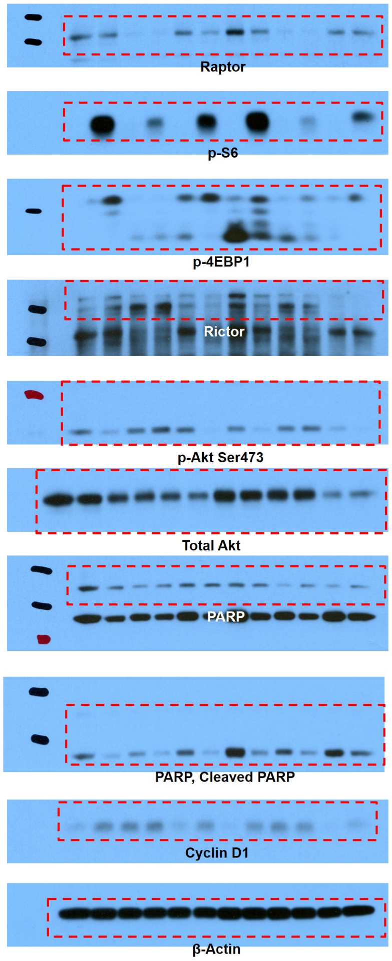

Figure 4d

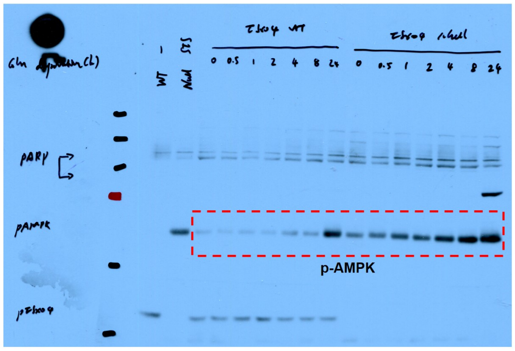

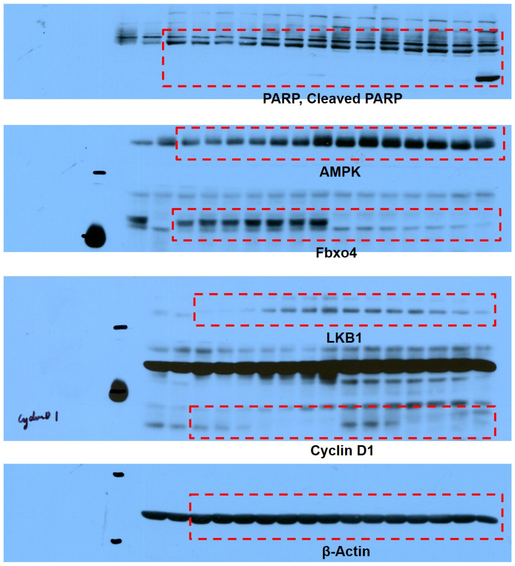

Supplementary Figure 23. Uncropped scans of the most important blots.

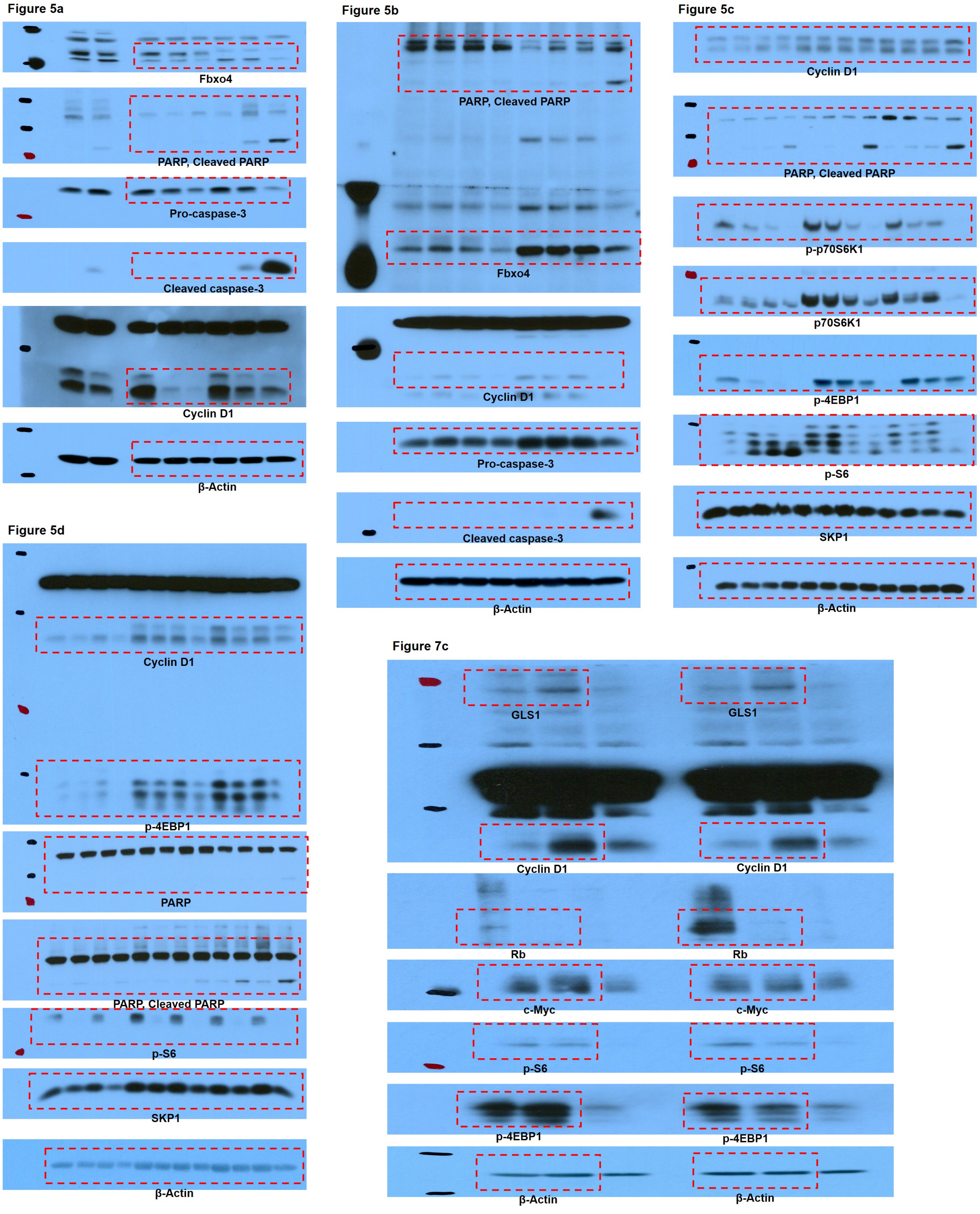

Supplementary Figure 23. Uncropped scans of the most important blots.

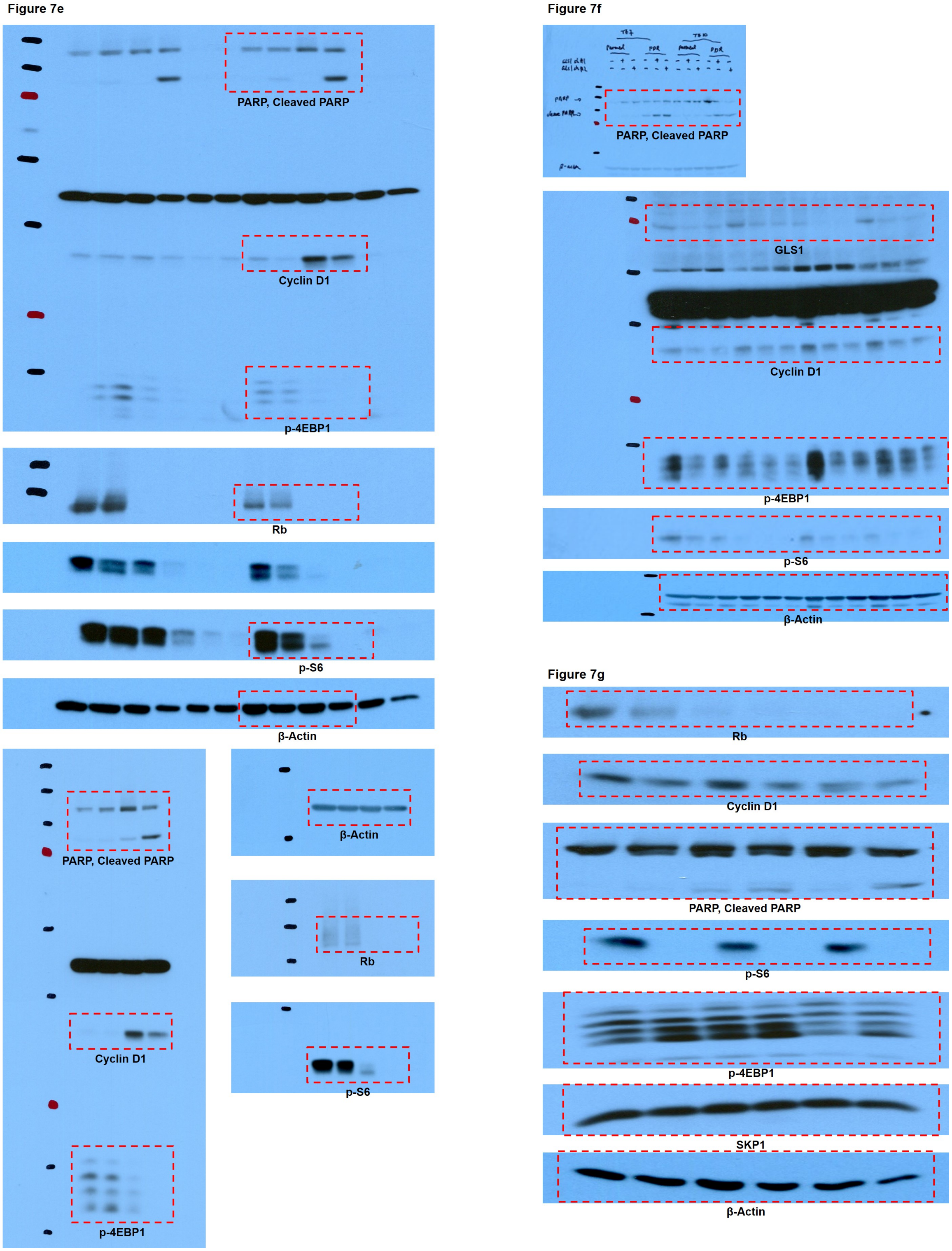

Supplementary Figure 23. Uncropped scans of the most important blots.

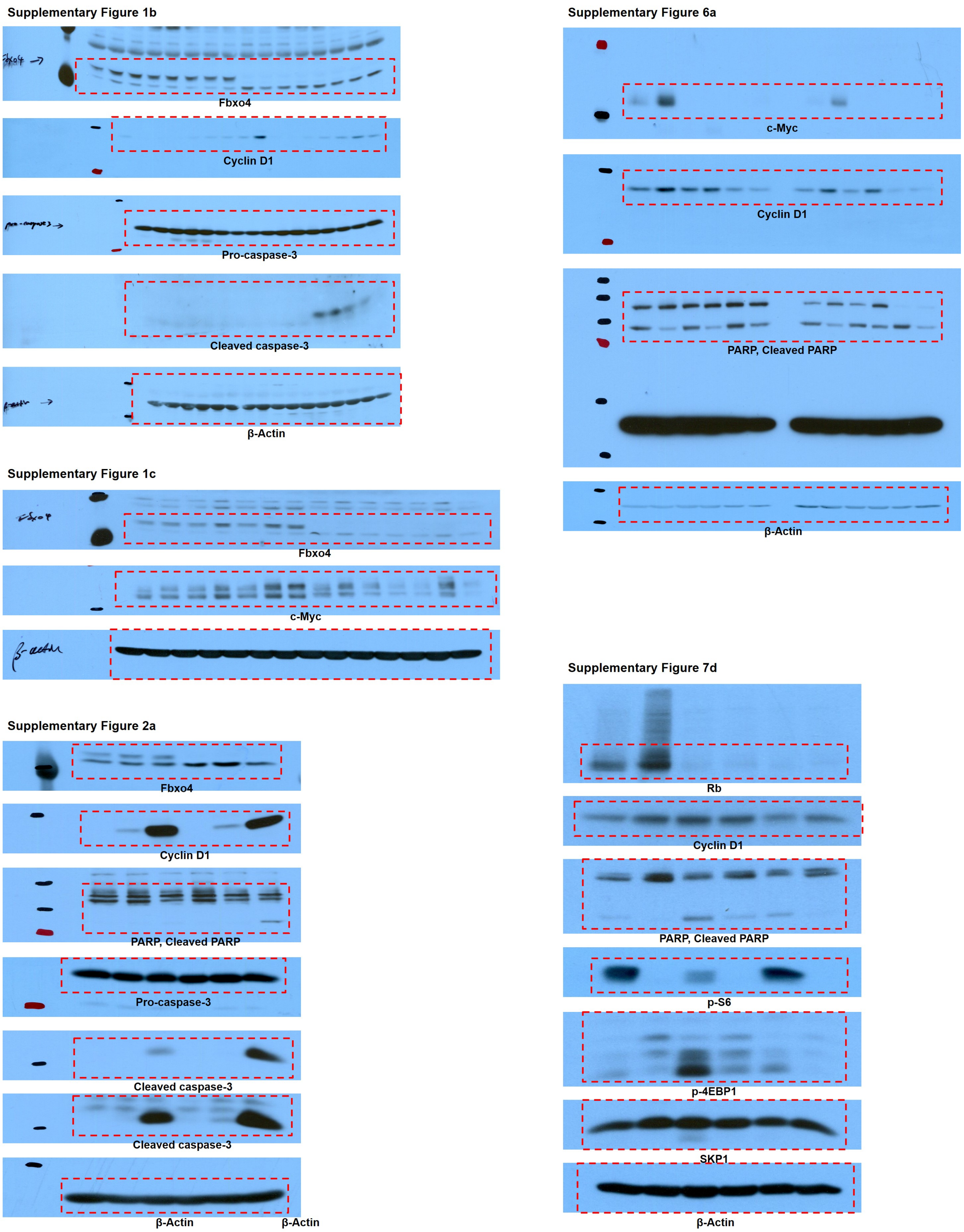

Supplementary Figure 23. Uncropped scans of the most important blots.

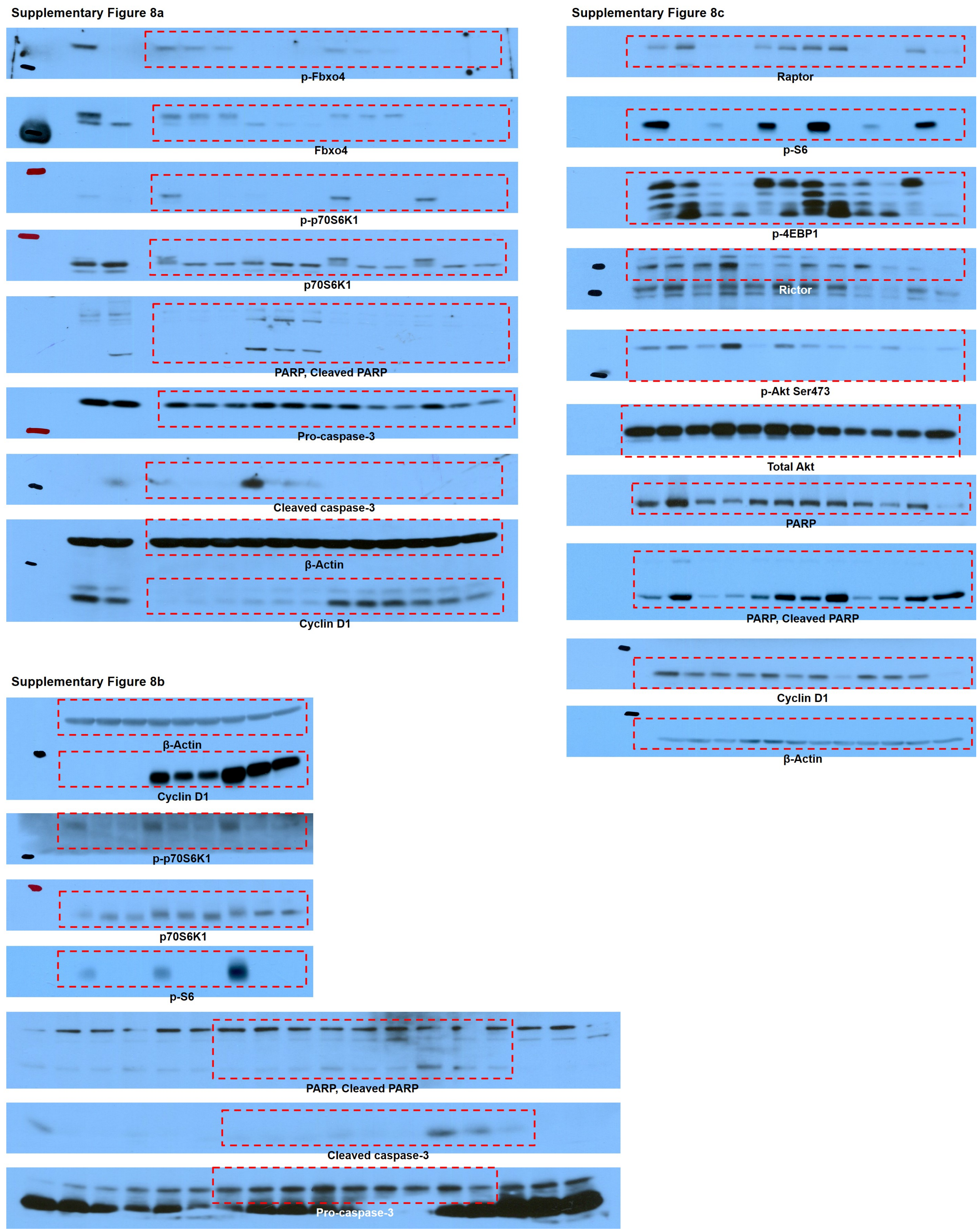

Supplementary Figure 23. Uncropped scans of the most important blots.

Supplementary Figure 10a

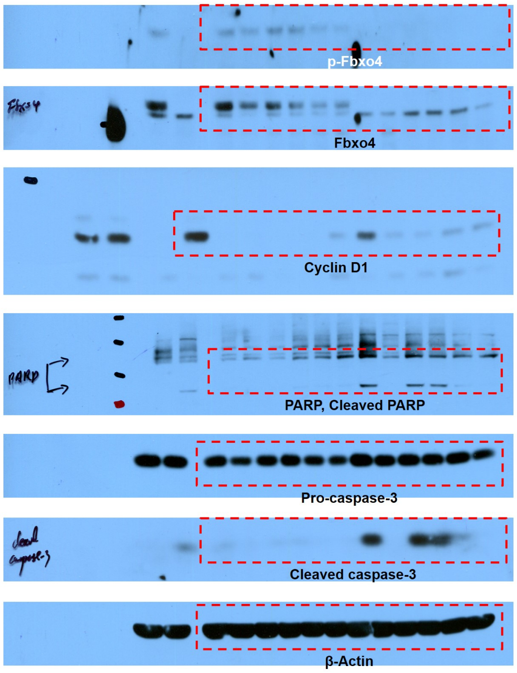

Supplementary Figure 10b

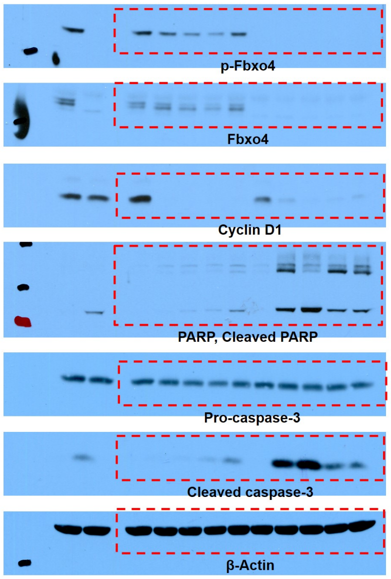

Supplementary Figure 10c

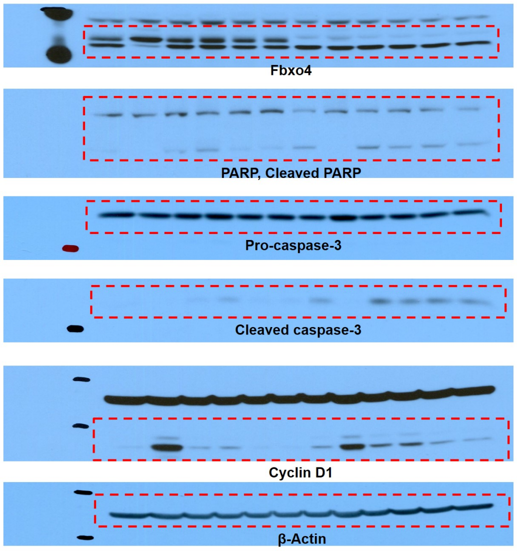

Supplementary Figure 11a

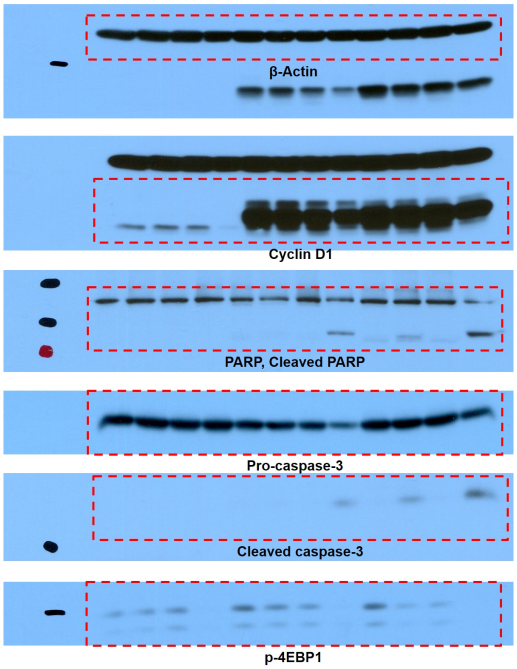

Supplementary Figure 23. Uncropped scans of the most important blots.

Supplementary Figure 11c

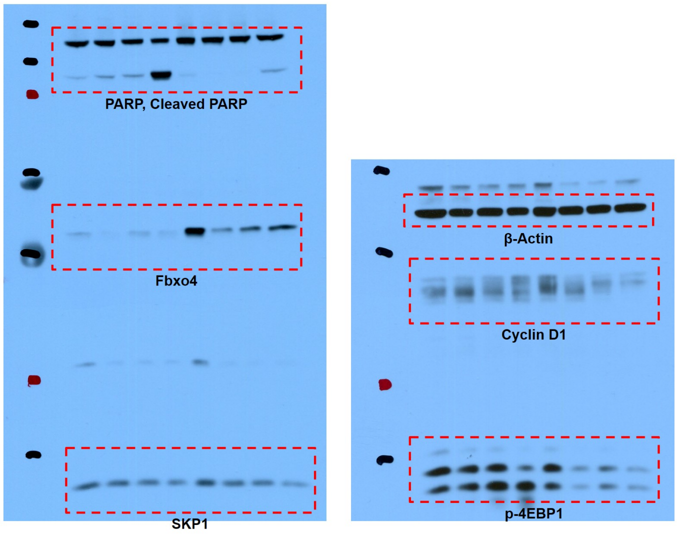

Supplementary Figure 11d

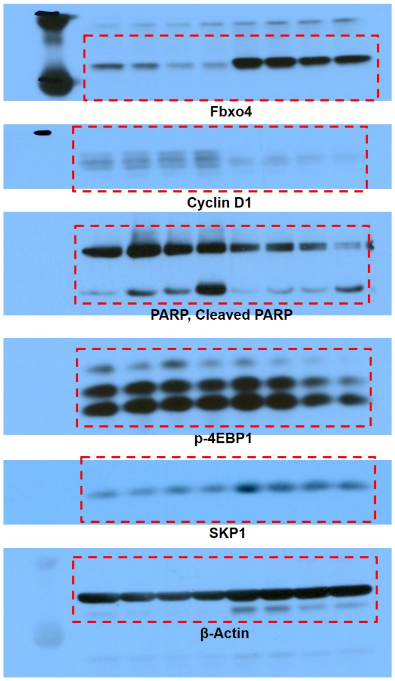

Supplementary Figure 18b

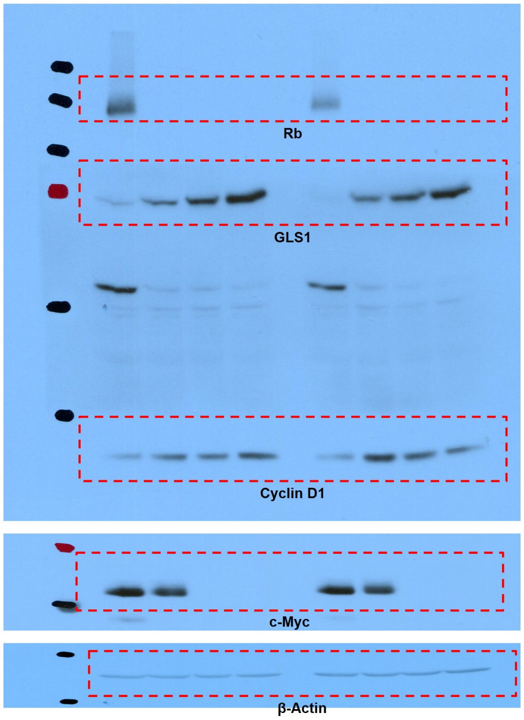

Supplementary Figure 18c

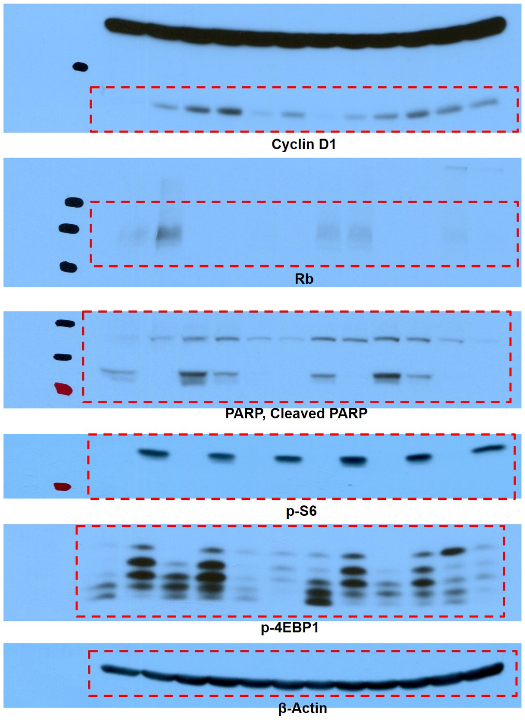

Supplementary Figure 23. Uncropped scans of the most important blots.

Supplementary Tables

Supplementary Table 1. KEGG Gene Sets Analysis of GSE100942 Using c2.cp.kegg.v6.1.symbols

NAME	SIZE	ES	NES	NOM p-val	FDR q-val	FWER p-val
KEGG_SPLICEOSOME	84	0.5378612	1.6993918	0.03187251	0.15339386	0.135
KEGG_AMINOACYL_TRNA_BIOSYNTHESIS	26	0.53128	1.5269792	0.0704501	0.5481249	0.587
KEGG_PROTEASOME	41	0.58322674	1.5164688	0.02794411	0.42280155	0.631
KEGG_BASE_EXCISION_REPAIR	31	0.62329453	1.4870512	0.05631068	0.4428937	0.731
KEGG_CELL_CYCLE	105	0.62945884	1.4644697	0.00963391	0.45459393	0.809
KEGG_STEROID_BIOSYNTHESIS	16	0.66282624	1.4639498	0.01202405	0.38285175	0.817
KEGG_NUCLEOTIDE_EXCISION_REPAIR	42	0.538583	1.4473726	0.15264188	0.38086003	0.89
KEGG_RNA_POLYMERASE	23	0.4774881	1.4297729	0.14949495	0.3924895	0.914
KEGG_HOMOLOGOUS_RECOMBINATION	25	0.70352167	1.4246688	0.02539063	0.35978484	0.919
KEGG_DNA_REPLICATION	32	0.7567036	1.4058506	0.09826589	0.37587827	0.931
KEGG_PROTEIN_EXPORT	17	0.5925224	1.3938651	0.06692913	0.37706858	0.947
KEGG_UBIQUITIN_MEDIATED_PROTEOLYSIS	108	0.3795273	1.3907284	0.10271318	0.35298175	0.947
KEGG_GALACTOSE_METABOLISM	23	0.41778016	1.3692155	0.08436214	0.38369626	0.979
KEGG_PYRIMIDINE_METABOLISM	73	0.4468327	1.3642222	0.1183432	0.36579323	0.979
KEGG_RNA_DEGRADATION	42	0.46477392	1.3535004	0.14285715	0.37080067	0.989
KEGG_OOCYTE_MEIOSIS	92	0.43804422	1.3388276	0.09741551	0.38631	0.995
KEGG_GLYCOSYLPHOSPHATIDYLINOSITOL_GPI_ANCHOR_BIOSYNTHESIS	19	0.43744335	1.3378816	0.07632094	0.3661311	0.995
KEGG_MISMATCH_REPAIR	22	0.65441	1.3344722	0.23314066	0.35512564	1
KEGG_P53_SIGNALING_PATHWAY	59	0.49190336	1.3287777	0.05813954	0.34691244	1
KEGG_BASAL_TRANSCRIPTION_FACTORS	31	0.4414479	1.2824267	0.20628683	0.435414	1
KEGG_N_GLYCAN_BIOSYNTHESIS	35	0.48550975	1.2477528	0.28070176	0.5037567	1
KEGG_BASAL_CELL_CARCINOMA	45	0.47446945	1.1838824	0.29045644	0.64760154	1
KEGG_SYSTEMIC_LUPUS_ERYTHEMATOSUS	102	0.45062748	1.1783574	0.33333334	0.633084	1
KEGG_NOTCH_SIGNALING_PATHWAY	34	0.37923783	1.177033	0.21588595	0.6110266	1
KEGG_PROGESTERONE_MEDIATED_OOCYTE_MATURATION	74	0.38361531	1.1731039	0.24497992	0.5976401	1
KEGG_BLADDER_CANCER	40	0.39138293	1.1228994	0.31764707	0.7045425	1
KEGG_CYSTEINE_AND_METHIONINE_METABOLISM	29	0.39300582	1.0764035	0.39756593	0.7993539	1
KEGG_GLYCOSPHINGOLIPID_BIOSYNTHESIS_LACTO_AND_NEOLACTO_SERIES	24	0.34328318	1.0692724	0.30532786	0.79030657	1
KEGG_ANTIGEN_PROCESSING_AND_PRESENTATION	81	0.23732044	1.0554267	0.3806818	0.7943593	1

KEGG_PATHWAYS_IN_CANCER	303	0.27860624	1.0264274	0.41035858	0.8457788	1
KEGG_SELENOAMINO_ACID_METABOLISM	18	0.4085816	1.0033495	0.47826087	0.874753	1
KEGG_RIG_I_LIKE_RECEPTOR_SIGNALING_PATHWAY	63	0.25585413	1.003219	0.4951267	0.8476052	1
KEGG_FRUCTOSE_AND_MANNOSE_METABOLISM	32	0.27515984	0.98334974	0.45742574	0.8687906	1
KEGG_ECM_RECEPTOR_INTERACTION	80	0.34625205	0.96138024	0.54375	0.89508045	1
KEGG_RENAL_CELL_CARCINOMA	68	0.27061468	0.9544492	0.49496982	0.8847114	1
KEGG_SMALL_CELL_LUNG_CANCER	82	0.29689443	0.95094997	0.5541667	0.8679198	1
KEGG_EPITHELIAL_CELL_SIGNALING_IN_HELICOBACTER_PYLORI_INFECTION	58	0.272336	0.94668984	0.59090906	0.85324186	1
KEGG_VIBRIO_CHOLERAE_INFECTION	46	0.24401388	0.94505185	0.62984496	0.8339459	1
KEGG_AMYOTROPHIC_LATERAL_SCLEROSIS_ALS	46	0.21937269	0.94473904	0.5437882	0.8140569	1
KEGG_LYSOSOME	105	0.2739554	0.9420734	0.5153374	0.7994746	1
KEGG_PRIMARY_IMMUNODEFICIENCY	34	0.42975596	0.9321816	0.5805825	0.80019504	1
KEGG_WNT_SIGNALING_PATHWAY	131	0.26322812	0.91968215	0.641129	0.80723804	1
KEGG_THYROID_CANCER	29	0.23195155	0.8767276	0.69793624	0.87170386	1
KEGG_MELANOGENESIS	89	0.2648372	0.8707144	0.75	0.86152387	1
KEGG_PANCREATIC_CANCER	69	0.24565984	0.86978376	0.748	0.84429145	1
KEGG_CITRATE_CYCLE_TCA_CYCLE	29	0.26545802	0.86668384	0.6895161	0.83194983	1
KEGG_GLIOMA	61	0.24597703	0.86426777	0.74493927	0.81915617	1
KEGG_ETHER_LIPID_METABOLISM	25	0.32193816	0.85696507	0.67241377	0.81506705	1
KEGG_GLYCOSAMINOGLYCAN_BIOSYNTHESIS_HEPARAN_SULFATE	20	0.28788182	0.84993917	0.73031497	0.81084555	1
KEGG_REGULATION_OF_AUTOPHAGY	31	0.2718954	0.8439529	0.64495796	0.8037318	1
KEGG_PATHOGENIC_ESCHERICHIA_COLI_INFECTION	42	0.2358273	0.8419502	0.7991968	0.7906145	1
KEGG_PURINE_METABOLISM	131	0.2258766	0.8251262	0.68356997	0.800493	1
KEGG_SPHINGOLIPID_METABOLISM	26	0.21924248	0.7988189	0.7883495	0.8243223	1
KEGG_PROSTATE_CANCER	85	0.22427446	0.76639026	0.88438135	0.8557531	1
KEGG_BIOSYNTHESIS_OF_UNSATURATED_FATTY_ACIDS	18	0.3007753	0.7609227	0.8117409	0.8471875	1
KEGG_HEDGEHOG_SIGNALING_PATHWAY	44	0.24740309	0.73404825	0.9185336	0.86734366	1
KEGG_GLUTATHIONE_METABOLISM	39	0.2751862	0.68140775	0.8745098	0.9094527	1
KEGG_PENTOSE_PHOSPHATE_PATHWAY	24	0.23464042	0.6562733	0.81578946	0.91443646	1
KEGG_OXIDATIVE_PHOSPHORYLATION	98	0.14800039	0.58093244	0.8086785	0.9515539	1

Supplementary Table 2. GSEA Analysis of GSE100942 for PENG_GLUTAMINE_DEPRIVATION_DN

NAME	SIZE	ES	NES	NOM p-val	FDR q-val	FWER p-val
PENG_GLUTAMINE_DEPRIVATION_DN	281	0.5033834	1.5255251	0.05846774	0.06451613	0.029

Supplementary Table 3. KEGG Gene Sets Analysis of GSE20347 Using c2.cp.kegg.v6.1.symbols

NAME	SIZE	ES	NES	NOM p-val	FDR q-val	FWER p-val
KEGG_SMALL_CELL_LUNG_CANCER	82	0.60083896	1.5112792	0.00819672	1	0.757
KEGG_HOMOLOGOUS_RECOMBINATION	25	0.74613047	1.4896505	0.0020284	1	0.815
KEGG_PATHWAYS_IN_CANCER	303	0.4621502	1.4632831	0.00208333	1	0.865
KEGG_ECM_RECEPTOR_INTERACTION	80	0.6945468	1.4566048	0.0310559	0.88940424	0.88
KEGG_BLADDER_CANCER	40	0.5742482	1.4546992	0.01629328	0.7215279	0.881
KEGG_PROGESTERONE_MEDIATED_OOCYTE_MATURATION	74	0.48452243	1.4521371	0.02282158	0.61329406	0.886
KEGG_CELL_CYCLE	105	0.6026979	1.4447355	0.04897959	0.55833924	0.902
KEGG_RNA_DEGRADATION	42	0.5368972	1.4204373	0.10865191	0.58732176	0.932
KEGG_PURINE_METABOLISM	131	0.3979154	1.4155347	0.05522683	0.5404721	0.938
KEGG_BASE_EXCISION_REPAIR	31	0.5832229	1.4065813	0.12151395	0.52005726	0.948
KEGG_PROTEASOME	41	0.61350846	1.3802122	0.17322835	0.56344265	0.963
KEGG_FOCAL_ADHESION	182	0.5121089	1.3690076	0.11434511	0.55809367	0.969
KEGG_PROSTATE_CANCER	85	0.43675244	1.3678819	0.04081633	0.51916057	0.969
KEGG_DNA_REPLICATION	32	0.76095086	1.3666668	0.08606558	0.48601222	0.969
KEGG_SPLICEOSOME	84	0.4808711	1.3604081	0.21975806	0.4721321	0.971
KEGG_SELENOAMINO_ACID_METABOLISM	18	0.5700955	1.3597511	0.11154599	0.4452587	0.972
KEGG_CYSTEINE_AND_METHIONINE_METABOLISM	29	0.52151835	1.3475213	0.15490197	0.45305493	0.977
KEGG_BASAL_TRANSCRIPTION_FACTORS	31	0.46380585	1.324099	0.1610338	0.49169382	0.98
KEGG_MELANOMA	68	0.44780892	1.3238244	0.08514851	0.46685928	0.98
KEGG_GLIOMA	61	0.38223347	1.3137153	0.07797271	0.46901214	0.983
KEGG_MISMATCH_REPAIR	22	0.7063976	1.3072473	0.14344262	0.462777	0.983

KEGG_P53_SIGNALING_PATHWAY	59	0.45875153	1.28979	0.1	0.48531038	0.988
KEGG_NON_SMALL_CELL_LUNG_CANCER	51	0.35240808	1.2697679	0.0761523	0.51730615	0.995
KEGG_NICOTINATE_AND_NICOTINAMIDE_METABOLISM	15	0.48176607	1.2692492	0.13778706	0.49719632	0.995
KEGG_RIG_I_LIKE_RECEPTOR_SIGNALING_PATHWAY	63	0.36520854	1.2305856	0.16699801	0.57605606	0.998
KEGG_OOCYTE_MEIOSIS	92	0.34539953	1.1963949	0.22156863	0.6460467	0.998
KEGG_BASAL_CELL_CARCINOMA	45	0.46237007	1.1872112	0.2704918	0.6473512	0.999
KEGG_N_GLYCAN_BIOSYNTHESIS	35	0.3777635	1.1694006	0.2602459	0.6723242	0.999
KEGG_NUCLEOTIDE_EXCISION_REPAIR	42	0.4481874	1.152892	0.352	0.69410175	1
KEGG_RNA_POLYMERASE	23	0.4588073	1.1467562	0.35714287	0.68747485	1
KEGG_CHRONIC_MYELOID_LEUKEMIA	71	0.31101954	1.1460354	0.23858921	0.66695225	1
KEGG_PYRIMIDINE_METABOLISM	73	0.40148392	1.1394376	0.34874758	0.66282296	1
KEGG_TGF_BETA_SIGNALING_PATHWAY	78	0.3870513	1.1390307	0.30039525	0.64364004	1
KEGG_NITROGEN_METABOLISM	22	0.42492476	1.0951015	0.31474105	0.7333508	1
KEGG_PANCREATIC_CANCER	69	0.30187157	1.0681324	0.28282827	0.7808154	1
KEGG_TOLL_LIKE_RECEPTOR_SIGNALING_PATHWAY	95	0.35048386	1.0662006	0.35578948	0.7642144	1
KEGG_CYTOKINE_CYTOKINE_RECEPTOR_INTERACTION	228	0.34534535	1.0546881	0.36401674	0.77374685	1
KEGG_ALANINE_ASPARTATE_AND_GLUTAMATE_METABOLISM	27	0.3737613	1.0538836	0.36831683	0.75529426	1
KEGG_COMPLEMENT_AND_COAGULATION_CASCADES	64	0.39023677	1.0510985	0.37960956	0.7424792	1
KEGG_REGULATION_OF_ACTIN_CYTOSKELETON	181	0.26784396	1.0470998	0.37263158	0.7335028	1
KEGG_EPITHELIAL_CELL_SIGNALING_IN_HELICOBACTER_PYLORI_INFECTION	58	0.35519758	1.0354358	0.4084507	0.7423099	1
KEGG_WNT_SIGNALING_PATHWAY	131	0.30394357	1.0266368	0.40681362	0.74532735	1
KEGG_TYPE_II_DIABETES_MELLITUS	42	0.27304962	1.0053351	0.4473161	0.7774164	1
KEGG_COLORECTAL_CANCER	60	0.29848194	1.0015484	0.4493927	0.76876074	1
KEGG_CHEMOKINE_SIGNALING_PATHWAY	161	0.32332677	1.001408	0.44731182	0.7520394	1
KEGG_RENAL_CELL_CARCINOMA	68	0.27251476	0.99545366	0.48076922	0.7492061	1
KEGG_NOD_LIKE_RECEPTOR_SIGNALING_PATHWAY	48	0.3871363	0.9934805	0.46750525	0.7373321	1
KEGG_VEGF_SIGNALING_PATHWAY	67	0.25037214	0.97718763	0.4821803	0.75695544	1
KEGG_AMYOTROPHIC_LATERAL_SCLEROSIS_ALS	46	0.25725695	0.97573435	0.49713194	0.74467486	1
KEGG_FC_GAMMA_R_MEDIATED_PHAGOCYTOSIS	82	0.259942	0.9246754	0.5995717	0.8396063	1
KEGG_AMINOACYL_TRNA_BIOSYNTHESIS	26	0.31211573	0.9137323	0.5882353	0.84655637	1
KEGG_ADIPOCYTOKINE_SIGNALING_PATHWAY	62	0.2535379	0.9120205	0.6300578	0.8339664	1
KEGG_NATURAL_KILLER_CELL_MEDIATED_CYTOTOXICITY	121	0.25788823	0.8873215	0.5893617	0.87051165	1
KEGG_ARRHYTHMOGENIC_RIGHT_VENTRICULAR_CARDIOMYOPATHY_ARVC	68	0.26279026	0.86967814	0.639485	0.8905307	1
KEGG_GALACTOSE_METABOLISM	23	0.29697353	0.85921216	0.6620553	0.89662963	1
KEGG_ABC_TRANSPORTERS	36	0.27215642	0.85030496	0.7227926	0.8974359	1

KEGG_UBIQUITIN_MEDIATED_PROTEOLYSIS	108	0.21840228	0.82608074	0.73239434	0.92763597	1
KEGG_MELANOGENESIS	89	0.24595398	0.81959975	0.7610063	0.92420304	1
KEGG_ACUTE_MYELOID_LEUKEMIA	56	0.23008123	0.8187737	0.80785125	0.91006196	1
KEGG_STEROID_BIOSYNTHESIS	16	0.35363293	0.8081507	0.78904665	0.9130337	1
KEGG_NOTCH_SIGNALING_PATHWAY	34	0.21353553	0.7897456	0.7348643	0.9289724	1
KEGG_MTOR_SIGNALING_PATHWAY	44	0.19973217	0.7872467	0.87148595	0.9181034	1
KEGG_APOPTOSIS	78	0.23755108	0.77643937	0.8378378	0.9204199	1
KEGG_PROTEIN_EXPORT	17	0.28648525	0.7679555	0.7076023	0.91893744	1
KEGG_CYTOSOLIC_DNA_SENSING_PATHWAY	47	0.25992128	0.7609045	0.7372881	0.9149839	1
KEGG_REGULATION_OF_AUTOPHAGY	31	0.17693204	0.7193552	0.861167	0.95836544	1
KEGG_HEDGEHOG_SIGNALING_PATHWAY	44	0.22416723	0.7161505	0.8778468	0.94816935	1
KEGG_GLUTATHIONE_METABOLISM	39	0.30743387	0.7126614	0.852071	0.9388392	1
KEGG_GLYCOSYLPHOSPHATIDYLINOSITOL_GPI_ANCHOR_BIOSYNTHESIS	19	0.25547138	0.707702	0.81439394	0.93080354	1
KEGG_HYPERTROPHIC_CARDIOMYOPATHY_HCM	76	0.21064377	0.688769	0.8801743	0.93829155	1
KEGG_RENIN_ANGIOTENSIN_SYSTEM	15	0.28154436	0.6661022	0.86734694	0.9466641	1
KEGG_CELL_ADHESION_MOLECULES_CAMS	115	0.21542	0.63634616	0.9686192	0.95688593	1
KEGG_SYSTEMIC_LUPUS_ERYTHEMATOSUS	102	0.19043681	0.5538107	0.9617706	0.9848914	1
KEGG_ANTIGEN_PROCESSING_AND_PRESENTATION	81	0.15277213	0.46351835	0.97336066	0.9920295	1

Supplementary Table 4. GSEA Analysis of GSE20347 for PENG_GLUTAMINE_DEPRIVATION_DN

NAME	SIZE	ES	NES	NOM p-val	FDR q-val	FWER p-val
PENG_GLUTAMINE_DEPRIVATION_DN	281	0.50012505	1.4435372	0.12038835	0.12038835	0.062

Supplementary Table 5. Hallmark Gene Sets Analysis of GSE100942 Using h.all.v6.1.symbols

NAME	SIZE	ES	NES	NOM p-val	FDR q-val	FWER p-val
HALLMARK_DNA_REPAIR	135	0.4307888	1.5358499	0.05186722	0.2944169	0.231
HALLMARK_MYC_TARGETS_V1	171	0.5355455	1.5030266	0.13871635	0.22406572	0.331
HALLMARK_MYC_TARGETS_V2	46	0.68100154	1.4826365	0.01464435	0.18403126	0.373
HALLMARK_SPERMATOGENESIS	117	0.49683934	1.4784535	0.02325581	0.14244156	0.375
HALLMARK_MITOTIC_SPINDLE	166	0.55819744	1.4611797	0.00849257	0.1415279	0.431
HALLMARK_MTORC1_SIGNALING	182	0.4569095	1.4599689	0.04752066	0.12097056	0.444
HALLMARK_E2F_TARGETS	167	0.74805516	1.4127096	0.00635593	0.1538755	0.569
HALLMARK_G2M_CHECKPOINT	176	0.69703484	1.3974775	0.02725367	0.152087	0.597
HALLMARK_GLYCOLYSIS	176	0.42345873	1.3560416	0	0.17563282	0.661
HALLMARK_UNFOLDED_PROTEIN_RESPONSE	104	0.41274795	1.3127109	0.18674698	0.22092712	0.776
HALLMARK_NOTCH_SIGNALING	26	0.34036228	1.1618803	0.218107	0.47265148	0.969
HALLMARK_HEDGEHOG_SIGNALING	35	0.4064567	1.1565981	0.15856236	0.4430317	0.969
HALLMARK_WNT_BETA_CATENIN_SIGNALING	37	0.35479745	1.0660331	0.35281837	0.58427227	0.992
HALLMARK_PEROXISOME	94	0.26075107	1.0517225	0.3319239	0.5697178	0.992
HALLMARK_INTERFERON_ALPHA_RESPONSE	77	0.43323758	1.0265052	0.46544716	0.58141756	0.997
HALLMARK_ANGIOGENESIS	34	0.41962942	0.96254927	0.5102041	0.67501676	0.997
HALLMARK_CHOLESTEROL_HOMEOSTASIS	60	0.26147357	0.93897474	0.5618861	0.68125546	1
HALLMARK_EPITHELIAL_MESENCHYMAL_TRANSITION	189	0.34536305	0.9062222	0.5860656	0.7090013	1
HALLMARK_PROTEIN_SECRETION	88	0.16960172	0.7004054	0.83433133	0.98879063	1
HALLMARK_INTERFERON_GAMMA_RESPONSE	171	0.25583893	0.6455057	0.8353909	0.97872883	1
HALLMARK_OXIDATIVE_PHOSPHORYLATION	188	0.11390571	0.47617602	0.91239315	0.9875101	1

Supplementary Table 6. Hallmark Gene Sets Analysis of GSE20347 Using h.all.v6.1.symbols

NAME	SIZE	ES	NES	NOM p-val	FDR q-val	FWER p-val
HALLMARK_SPERMATOGENESIS	117	0.48719388	1.4880008	0.01807229	0.7483004	0.372
HALLMARK_G2M_CHECKPOINT	176	0.67332107	1.4563006	0.02713178	0.49793926	0.44
HALLMARK_ANGIOGENESIS	34	0.72443247	1.4455006	0.03501945	0.36677426	0.465
HALLMARK_E2F_TARGETS	167	0.7133889	1.427559	0.05598456	0.3254115	0.516
HALLMARK_MYC_TARGETS_V1	171	0.5371301	1.4103597	0.19885278	0.30219287	0.555
HALLMARK_MYC_TARGETS_V2	46	0.63685215	1.3855946	0.15037593	0.3041315	0.599
HALLMARK_EPITHELIAL_MESENCHYMAL_TRANSITION	189	0.7149435	1.3687184	0.06451613	0.2952076	0.636
HALLMARK_DNA_REPAIR	135	0.42442423	1.3340472	0.1764706	0.33268628	0.702
HALLMARK_MITOTIC_SPINDLE	166	0.45254612	1.3027898	0.11523438	0.36924365	0.776
HALLMARK_UNFOLDED_PROTEIN_RESPONSE	104	0.42034298	1.3016204	0.17142858	0.3344757	0.778
HALLMARK_MTORC1_SIGNALING	182	0.4339273	1.269795	0.1904762	0.3669077	0.838
HALLMARK_COAGULATION	132	0.4273528	1.2556125	0.16007534	0.36570895	0.864
HALLMARK_HEDGEHOG_SIGNALING	35	0.460401	1.245114	0.19075145	0.3589455	0.874
HALLMARK_APICAL_JUNCTION	180	0.35056978	1.1502165	0.23416507	0.5244455	0.954
HALLMARK_GLYCOLYSIS	176	0.361889	1.1303022	0.2627451	0.53435504	0.964
HALLMARK_UV_RESPONSE_UP	150	0.34497172	1.092986	0.2744361	0.58311003	0.979
HALLMARK_KRAS_SIGNALING_UP	191	0.37934256	1.0830822	0.2933071	0.56884366	0.983
HALLMARK_INTERFERON_ALPHA_RESPONSE	77	0.52915335	1.0545579	0.45039684	0.59629357	0.988
HALLMARK_WNT_BETA_CATENIN_SIGNALING	37	0.34381878	0.9824552	0.5030675	0.72089374	0.998
HALLMARK_CHOLESTEROL_HOMEOSTASIS	60	0.33693036	0.97543406	0.4773585	0.6997811	0.998
HALLMARK_INFLAMMATORY_RESPONSE	185	0.368945	0.973382	0.48508945	0.67079914	0.998
HALLMARK_UV_RESPONSE_DN	135	0.36464074	0.97083473	0.48991936	0.6455642	0.998
HALLMARK_TGF_BETA_SIGNALING	49	0.3660519	0.9521238	0.5294118	0.6521895	0.999
HALLMARK_INTERFERON_GAMMA_RESPONSE	171	0.39591375	0.9190325	0.5551181	0.68494904	1
HALLMARK_HYPOXIA	182	0.30072927	0.8971757	0.60536397	0.69917005	1
HALLMARK_TNFA_SIGNALING_VIA_NFKB	185	0.3249574	0.8071502	0.7346535	0.83164096	1
HALLMARK_COMPLEMENT	181	0.27118385	0.7987521	0.79476863	0.81496185	1
HALLMARK_IL6_JAK_STAT3_SIGNALING	85	0.26081663	0.7115504	0.87234044	0.9055163	1
HALLMARK_ALLOGRAFT_REJECTION	191	0.21985768	0.59929836	0.9049505	0.95763576	1

Supplementary Table 7. GMX Database for Qie_Glutamine Metabolism

Qie_Glutamine Metabolism							
na	ALDH4A1	SUCLA2	NDUFS3	NDUFB3	UQCR11	COX17	ATP6V1B1
GOT1	GLUL	SDHA	NDUFS4	NDUFB4	COX10	ATP5E	ATP6V1B2
GOT2	CAD	SDHB	NDUFS5	NDUFB5	COX3	ATP5D	ATP6V1E1
IL4I1	GLS2	SDHC	NDUFS6	NDUFB6	COX1	ATP5B	ATP6V1E2
DDO	GLS1	SDHD	NDUFS7	NDUFB7	COX2	ATP5C1	ATP6V0C
ASNS	CPS1	FH	NDUFS8	NDUFB8	COX4I1	ATP5A1	ATP6V0B
NIT2	GFPT1	MDH1	NDUFV1	NDUFB9	COX4I2	ATP5O	TCIRG1
GPT	GFPT2	MDH2	NDUFV2	NDUFB10	COX5A	ATP8	ATP6V0A2
GPT2	PPAT	PC	NDUFV3	NDUFC1	COX5B	ATP5G1	ATP6V0A4
AGXT	CS	PCK1	NDUFA1	NDUFC2	COX6A1	ATP5G2	ATP6V0A1
AGXT2	ACLY	PCK2	NDUFA2	SDHC	COX6A2	ATP5G3	ATP6V1D
ASS1	ACO1	PDHA1	NDUFA3	SDHD	COX6B2	ATP6	ATP6V0E2
ASL	ACO2	PDHA2	NDUFA4	SDHA	COX6B1	ATP5F1	ATP6V0E1
ADSSL1	IDH1	PDHB	NDUFA4L2	SDHB	COX6C	ATP5I	ATP6V1G3
ADSS	IDH2	DLAT	NDUFA5	UQCRFS1	COX7A1	ATP5J	ATP6V1G2
ADSL	IDH3A	ND1	NDUFA6	CYTB	COX7A2	ATP5J2	ATP6V1G1
ASPA	IDH3B	ND2	NDUFA7	CYC1	COX7A2L	ATP5H	ATP6V1H
ACY3	IDH3G	ND3	NDUFA8	UQCRC1	COX7B	ATP5L	ATP6AP1
GAD1	OGDH	ND4	NDUFA9	UQCRC2	COX7B2	ATP6V0D2	ATP4A
GAD2	OGDHL	ND4L	NDUFA10	UQCRHL	COX7C	ATP6V0D1	ATP4B
ABAT	DLST	ND5	NDUFAB1	UQCRH	COX8A	ATP6V1F	ATP12A
ALDH5A1	DLD	ND6	NDUFA11	UQCRB	COX8C	ATP6V1C2	PPA2
GLUD1	SUCLG1	NDUFS1	NDUFB1	UQCRQ	COX11	ATP6V1C1	PPA1
GLUD2	SUCLG2	NDUFS2	NDUFB2	UQCR10	COX15	ATP6V1A	LHPP

Supplementary Methods

Antibodies and chemicals

Rabbit anti-pSer11/12 and total Fbxo4 antibodies (YenZym Antibodies), mouse anti-cyclin D1 antibody (D1-72-13G), mouse anti-human cyclin D1 antibody (Calbiochem), rabbit anti- PARP, caspase-3 and cleaved caspase-3 antibodies (Cell Signaling), rabbit anti- c-Myc and pSer780 Rb antibodies (Santa Cruz), mouse anti- SKP1 and Rb antibodies (BD Transduction Laboratories™), rabbit anti- pSer473 and total Akt, pThr1462 and total TSC2, p70S6K1, pSer235/236 S6, pThr37/46 4E-BP1, pThr172 and total AMPK, LKB1 antibodies and mouse anti- p-p70S6K1 antibody (Cell Signaling), rabbit anti- Raptor and Rictor antibodies (Bethyl Laboratories), mouse anti- GLS1 (Abcam), and mouse anti- β-actin antibody (Sigma).

200mM Gln solution from Gibco; Asparagine (Asn), Dimethyl α-ketoglutarate (DM-α-KG), (-)-Epigallocatechin gallate (EGCG), N-Acetyl-L-cysteine (NAC), L-Ascorbic acid (Vitamin C, VitC), Staurosporine (STS), Oligomycin, Carbonyl cyanide 4-(trifluoromethoxy) phenylhydrazone (FCCP), Antimycin A and Rotenone, Phenformin, sodium citrate, and hydroxy propyl-β-cyclodextrin from Sigma; Palbociclib (PD-0332991), CB-839 and Rad001 from Selleck Chemicals; Metformin and Rapamycin from EMD Millipore Calbiochem™.

Flow cytometry (FACS) analysis

Upon relative treatment, cells were trypsinized, washed with 1× cold PBS, stained with PE conjugated Annexin V (BD Pharmingen™), and sorted by BD FACSVerse (BD Biosciences-US). Cells treated with staurosporine (STS) were used as positive controls, and data were analyzed using FlowJo software (Tree Star, Inc).

For cell cycle analysis, cells were trypsinized, washed with 1× cold PBS after relative treatment. Then, cells were stained with 10 μg/mL Propidium Iodide (PI) containing 100 μg/mL RNaseA before FACS analysis.

Microarray data normalization

Four cDNA microarray datasets were downloaded from Gene Expression Omnibus (https://www.ncbi.nlm.nih.gov/geo/). GSE100942 and GSE20347 include both ESCC and normal tissues; GSE40513 compares palbociclib and vehicle treated mouse breast cancer V720 cells; GSE84597 compares *CDK4/6* siRNA and control siRNA transfected human HCT116 cells. These GEO datasets were uploaded to the R-Project Bioconductor and standardized using the Robust Multiarray Average (RMA) method[1]. The signal intensities were shown in a Log^2 scale and the normalization of gene expression was evaluated using the LIMMA package from Bioconductor[2].

In this study, R-Project Bioconductor (ver. 3.4.2, 09/28/2017) was utilized to normalize the affymetrix data. These codes are listed below:

```
# Load the script from the Internet and install bioconductor
source("http://bioconductor.org/biocLite.R")
# Then, download and install each package
biocLite("affy")
biocLite("oligo")
biocLite("limma")
# Load the Affymetrix library
library(affy)
```

Change the directory, read the relative CEL files in it, and normalize the data

```
data <- ReadAffy()

eset <- rma(data)
```

Finally, save the data to an output file that can be used by GSEA analysis (Data will be log2 transformed and normalized)

```
write.exprs(eset,file="data.txt")
```

Gene Set Enrichment Analysis (GSEA)

The normalized datasets were processed according to the instructions from GSEA website[3] (http://software.broadinstitute.org/gsea/index.jsp). Thereafter, GSEA was applied to analyze gene signatures using two datasets - Hallmark gene sets (h.all.v6.1.symbols), Curated gene sets (c2.cp.kegg.v6.1.symbols) as well as PENG_GLUTAMINE_DEPRIVATION_DN (http://software.broadinstitute.org/gsea/msigdb/geneset_page.jsp?geneSetName=PENG_GLUTAMINE_DEPRIVATION_DN) following GSEA analysis instructions. Normalized enrichment score (NES) was applied to rank the geneset enrichment by comparing ESCC vs normal tissues. A false discovery rate q-value (FDR q-value) was calculated to estimate the probability of a false positive finding. In addition, a family wise-error rate p-value (FWER p-value) was utilized to estimate the probability of a false positive finding for NES.

Composition of GSEA Dataset for Molecular Signatures Database

To analyze genes related to Gln metabolism, the Molecular Signatures Database was composed by downloading KEGG genes for Alanine, aspartate and glutamate metabolism, Citrate cycle (TCA cycle) and Oxidative phosphorylation from Metabolic gEne RApid Visualizer (http://merav.wi.mit.edu/SearchByGenes.html). Please refer to **Supplementary Table**

7 for detailed gene list. The *.gmx database was generated following instructions on the website: http://software.broadinstitute.org/cancer/software/gsea/wiki/index.php/Data_formats.

Oncomine Analysis

Oncomine analysis was performed using the online cancer microarray database (www.oncomine.org). The expression of *GLS1* was analyzed at mRNA levels based on the comparison between ESCC and their normal counterparts.

SurvExpress Survival Analysis

SurvExpress database[4] (http://bioinformatica.mty.itesm.mx:8080/Biomatec/SurvivaX.jsp) was utilized to analyze the correlation between the expression of Gln metabolism genes with survival of patients suffering from esophageal cancer or Head and Neck squamous cell carcinoma. The Gln metabolism gene list included *GLS1*, *GLS2*, *GLUL*, *GLUD1*, *GLUD2*, *ASNS*, *GOT1*, *GOT2*, *GPT* and *GPT2*.

Determination of EC50 and synergistic effects

Cells were plated in triplicate in 96-well plate at a density of 1×10^3 cells per well. The following day, cells were treated with different concentrations of CB-839 and/or metformin. Forty-eight hour post-treatment, cell numbers were examined using the CyQUANT® NF Cell Proliferation Assay Kit (Invitrogen, Grand Island, NY). The EC50s were determined by Hill equation.

Clonogenic assay

Cells were split, and 2.5×10^3 cells were plated in 60 mm dishes. The following day, cells were treated with CB-839 and/or metformin. Twenty-four hour later, fresh medium were added. Giemsa stain was applied to detect the colonies.

^1H NMR spectroscopy analysis

Tissue samples were flash frozen in liquid nitrogen to halt metabolism. Tissues were then stored at −80°C. A 2-step tissue extraction was used, based on the Bligh Dyer method[5, 6], samples were extracted in batches of six. All tumour and liver samples were weighed prior to tissue extraction in order to determine $MeOH:H_2O$ and $CHCl_3:H_2O$ ratios used for tissue extraction. All samples were homogenized on ice using a hand-held homogenizer for 2× thirty-second-pulse. Following homogenization the samples were plunged into liquid nitrogen and stored at −80°C until all homogenizations within the batch were completed. Homogenates were transferred to a 5 ml glass centrifuge tube containing $CHCl_3:H_2O$ and vortexed for 1 minute and incubated on ice for a further 9 minutes. Separation of the polar and non-polar phases was achieved by centrifugation at 2,000 g at 4°C for 5 minutes. Polar and non-polar phases were carefully extracted using glass pipettes and transferred to 1.5 ml Eppendorf tubes for the polar fraction and 2.0 ml glass vials for non-polar fraction. Polar fractions were dried using a rotor evaporator under vacuum for 3 hours and stored at −80°C.

Dried extracts were reconstituted in 525 µl ^2H_2O and phosphate buffer (100 mM NaH_2PO_4/Na_2HPO_4 prepared using ^2H_2O, pH 7.2) containing 0.1% sodium azide and 1 mM sodium 3-(trimethylsilyl)-2,2,3,3-d_4-propionate (TMSP). Reconstituted samples were stored at 4°C prior to NMR analysis. A quality control (QC) sample was generated for each tissue type by pooling 25 µl from each of the reconstituted samples and 1 mM formate added as an additional internal standard.

All analyses were conducted on a Bruker Avance II 600 MHz spectrometer equipped with a room temperature TXI probe calibrated to 300 K. A pooled sample for each tissue type was used to create a three-dimensional shim set to ensure magnetic field homogeneity and

provide better NMR line-shapes. One-dimensional ^{1}H NMR spectra were obtained using a NOESY-presaturation pulse sequence for solvent suppression with an NOE mixing time of 60 ms. All spectra were recorded with a spectral width of 12 KHz, an acquisition time of 2.7 s, a relaxation delay of 5.0 s, 64K data points were collected for 128 scans. The resultant datasets were zero filled to 131K points and then Fourier transformed with a weighted exponential line-broadening factor of 0.5 Hz. The spectra were manually phased and base line corrected using Chenomx NMR suite version 8.3 (Chenomx Inc, Edmonton, Alberta, Canada; chenomx.com). A shim and line shape correction was automatically calculated and applied as part of the Chenomx processing suite, this ensured that all line shapes for each dataset were normalized to the TMSP internal standard peak.

Metabolites were identified and quantified using the Chenomx compound library. Due to signal overlap specific spectral regions of glucose (Glc), Gln and glutamate (Glu) were used for quantification that had little to no signal overlap (Glc: 3.7, 3.5, 3.45 and 3.2 ppm; Gln: 2.5 and 2.4 ppm; Glu: 2.35, 2.3 and 2.0 ppm). Spectral processing and data analyses were repeated in triplicate, by a single user with no knowledge of the study parameters, to ensure the highest degree of accuracy. Metabolite concentrations were adjusted to account for pooled sample donations, amount of material used for the extraction and loss during transfer steps.

RNA extraction and qRT-PCR

Parental and PDR TE7 and TE10 cells were cultured for 24 h and then washed with 1× ice-cold PBS. Total RNA was extracted using RNeasy Mini Kit. One µg total RNA from each sample was reverse transcribed using SuperScript III Reverse Transcriptase according to the manufacturer's protocol. The cDNA (1:15 dilution) was used for quantitative analysis using predesigned and validated TaqMan probes: *GLS1* (Hs01014020_m1) and *GLS2*

(Hs00998733_m1). qRT-PCR was performed as following: 2 min pre-incubation at 50°C, 10 min denaturation at 95°C, and 40 cycles of 15 s denaturation at 95°C, 1 min annealing and extension at 60°C. Relative RNA levels were normalized to *β-actin* (Hs01060665_g1). Relative mRNA levels were calculated using 2ˆ(-delta delta CT) method.

cBioportal TCGA Analysis

The gene mutation and copy number alterations (CNA) of *CCND1* and *Rb1* were analyzed using RNASeqV2 data from The Cancer Genome Atlas (TCGA)[7, 8] (www.cbioportal.org).

Supplementary References

1. Irizarry RA, Bolstad BM, Collin F, Cope LM, Hobbs B, Speed TP. Summaries of Affymetrix GeneChip probe level data. *Nucleic Acids Res* **31**, e15 (2003).

2. Smyth GK. Linear models and empirical bayes methods for assessing differential expression in microarray experiments. *Stat Appl Genet Mol Biol* **3**, Article3 (2004).

3. Subramanian A*, et al.* Gene set enrichment analysis: a knowledge-based approach for interpreting genome-wide expression profiles. *Proc Natl Acad Sci U S A* **102**, 15545-15550 (2005).

4. Aguirre-Gamboa R*, et al.* SurvExpress: an online biomarker validation tool and database for cancer gene expression data using survival analysis. *PLoS One* **8**, e74250 (2013).

5. Bligh EG, Dyer WJ. A rapid method of total lipid extraction and purification. *Can J Biochem Physiol* **37**, 911-917 (1959).

6. Wu H, Southam AD, Hines A, Viant MR. High-throughput tissue extraction protocol for NMR- and MS-based metabolomics. *Anal Biochem* **372**, 204-212 (2008).

7. Cerami E*, et al.* The cBio cancer genomics portal: an open platform for exploring multidimensional cancer genomics data. *Cancer Discov* **2**, 401-404 (2012).

8. Gao J*, et al.* Integrative analysis of complex cancer genomics and clinical profiles using the cBioPortal. *Sci Signal* **6**, pl1 (2013).
