# Supplementary material for: Targeting glutamine-addiction and overcoming CDK4/6 inhibitor resistance in human esophageal squamous cell carcinoma
Source: Nat Commun. 2019 Mar 21;10:1296. doi: 10.1038/s41467-019-09179-w (PMC6428878; doi:10.1038/s41467-019-09179-w)
Supplement: Supplementary file 3 — Reporting Summary [file 41467_2019_9179_MOESM3_ESM.pdf]

nature research

Corresponding author(s): J. Alan Diehl

Last updated by author(s): Feb 4, 2019

Reporting Summary

Nature Research wishes to improve the reproducibility of the work that we publish. This form provides structure for consistency and transparency in reporting. For further information on Nature Research policies, see Authors & Referees and the Editorial Policy Checklist.

Statistics

For all statistical analyses, confirm that the following items are present in the figure legend, table legend, main text, or Methods section.

n/a	Confirmed	
☐	☒	The exact sample size (*n*) for each experimental group/condition, given as a discrete number and unit of measurement
☐	☒	A statement on whether measurements were taken from distinct samples or whether the same sample was measured repeatedly
☐	☒	The statistical test(s) used AND whether they are one- or two-sided<br>*Only common tests should be described solely by name; describe more complex techniques in the Methods section.*
☐	☒	A description of all covariates tested
☐	☒	A description of any assumptions or corrections, such as tests of normality and adjustment for multiple comparisons
☐	☒	A full description of the statistical parameters including central tendency (e.g. means) or other basic estimates (e.g. regression coefficient) AND variation (e.g. standard deviation) or associated estimates of uncertainty (e.g. confidence intervals)
☒	☐	For null hypothesis testing, the test statistic (e.g. *F*, *t*, *r*) with confidence intervals, effect sizes, degrees of freedom and *P* value noted<br>*Give P values as exact values whenever suitable.*
☒	☐	For Bayesian analysis, information on the choice of priors and Markov chain Monte Carlo settings
☒	☐	For hierarchical and complex designs, identification of the appropriate level for tests and full reporting of outcomes
☒	☐	Estimates of effect sizes (e.g. Cohen's *d*, Pearson's *r*), indicating how they were calculated

Our web collection on statistics for biologists contains articles on many of the points above.

Software and code

Policy information about availability of computer code

Data collection

No software was used.

Data analysis

We used R-Project Bioconductor (ver. 3.4.2, 09/28/2017) to normalize the affymetrix data. The code is listed in the following:

```
# Load the script from the internet and install bioconductor
source("http://bioconductor.org/biocLite.R")

# Then, download and install each package
biocLite("affy")
biocLite("oligo")
biocLite("limma")

# Load the Affymetrix library
library(affy)

# Change the directory, read the relative CEL files in it, and normalize the data
data <- ReadAffy()
eset <- rma(data)

# Finally, save the data to an output file that can be used by GSEA analysis (Data will be log2 transformed and normalized)
write.exprs(eset,file="data.txt")
```

For manuscripts utilizing custom algorithms or software that are central to the research but not yet described in published literature, software must be made available to editors/reviewers. We strongly encourage code deposition in a community repository (e.g. GitHub). See the Nature Research guidelines for submitting code & software for further information.

Data

Policy information about availability of data

All manuscripts must include a data availability statement. This statement should provide the following information, where applicable:

- Accession codes, unique identifiers, or web links for publicly available datasets
- A list of figures that have associated raw data
- A description of any restrictions on data availability

The authors declare that all the data supporting the findings of this study are available with the article and its Supplementary Information files and from the corresponding author on reasonable request. In addition, the genome-wise data referenced during the study are available in a public repository from the NCBI Gene Expression Omnibus (https://www.ncbi.nlm.nih.gov/geo/) with GEO Accession #: GSE100942, GSE20347, GSE40513 and GSE84597, respectively.

Field-specific reporting

Please select the one below that is the best fit for your research. If you are not sure, read the appropriate sections before making your selection.

☒ Life sciences ☐ Behavioural & social sciences ☐ Ecological, evolutionary & environmental sciences

For a reference copy of the document with all sections, see nature.com/documents/nr-reporting-summary-flat.pdf

Life sciences study design

All studies must disclose on these points even when the disclosure is negative.

Sample size	For all cell-based work, three-distinct sample collections were performed in order to achieve high repeating efficiency and sufficient biostatistic significance. For all mice-based studies, 8 tumour samples were collected for final analysis; scientifically, this number can produce high confident statistical results.
Data exclusions	No data was excluded from the final analysis.
Replication	All attempts at replication were successful.
Randomization	All the cells and mice were randomly allocated into different groups.
Blinding	The investigators were blinded to group allocation during data collection and/or analysis.

Reporting for specific materials, systems and methods

We require information from authors about some types of materials, experimental systems and methods used in many studies. Here, indicate whether each material, system or method listed is relevant to your study. If you are not sure if a list item applies to your research, read the appropriate section before selecting a response.

Materials & experimental systems

n/a	Involved in the study	
☐	☒	Antibodies
☐	☒	Eukaryotic cell lines
☒	☐	Palaeontology
☐	☒	Animals and other organisms
☒	☐	Human research participants
☒	☐	Clinical data

Methods

n/a	Involved in the study	
☒	☐	ChIP-seq
☐	☒	Flow cytometry
☒	☐	MRI-based neuroimaging

Antibodies

Antibodies used	Rabbit anti-pSer11/12 and total Fbxo4 antibodies (YenZym Antibodies), mouse anti-cyclin D1 antibody (D1-72-13G), mouse anti-human cyclin D1 (cat. CC12) antibody (Calbiochem), rabbit anti- PARP (cat. 9542), caspase-3 (cat. 9665) and cleaved caspase-3 (cat. 9661) antibodies (Cell Signaling), rabbit anti- c-Myc (cat. sc-40) and pSer780 Rb (cat. sc-12901) antibodies (Santa Cruz), mouse anti- SKP1 (cat. 610530) and Rb (cat. 554136) antibodies (BD Transduction Laboratories™), rabbit anti- pSer473 (cat. 4058) and total Akt (cat. 9272), pThr1462 (cat. 3617) and total TSC2 (cat. 4308), p70S6K1 (cat. 9206), pSer235/236 S6 (cat. 4858), pThr37/46 4E-BP1 (cat. 2855), pThr172 (cat. 2535) and total AMPK (cat. 2532), LKB1 (cat. 3047) antibodies and mouse anti- p-p70S6K1 (cat. 2708) antibody (Cell Signaling), rabbit anti- Raptor (cat. A310-161A) and Rictor (cat. A300-459A) antibodies (Bethyl Laboratories), mouse anti- GLS1 (cat. ab60709) (Abcam), and mouse anti- β-actin (cat. A5316) antibody (Sigma). Mouse anti-Ki-67 (IR626) antibody (Agilent Technologies).
Validation	The commercial antibodies have relative validation statement indicating they can be used for these species on the

manufacturers' websites. All home-made antibodies have validation from previous publications: Rabbit anti-pSer11/12 and total Fbxo4 antibodies (Nat Commun. 2017 Nov 16;8(1):1534.); mouse anti-cyclin D1 antibody (Cancer Cell. 2008 Jul 8;14(1):68-78.).

Eukaryotic cell lines

Policy information about cell lines

Cell line source(s)	NIH3T3 and 293T cells were purchased from ATCC. TE1, TE7, TE8, TE10 and TE15 ESCC cells were kindly provided by Dr. Tetsuro Nishihara who established these cell lines. Fbxo4 +/+ and −/− MEFs, TE7 PDR and TE10 PDR cells were established by our lab.
Authentication	NIH3T3 and 293T cells have the authentication information from ATCC. The earliest frozen stocks of all ESCC cell lines (TE1, TE7, TE8, TE10 and TE15) have been stored at the Cell Culture Core of the University of Pennsylvania. All cells were authenticated by short tandem repeat analysis for highly polymorphic microsatellites FES/FPS, vWA31, D22S417, D10S526 and D5S592 as performed by the Cell Culture Core to validate the identity of cells by comparing the earliest stocks with those grown more than 8-12 passages. We established the MEF cells through a 3T9 protocol, in which cells were passaged and spontaneously transformed to proliferative cells after passage 9; the MEFs were identified through genotyping using PCR methods. TE7 PDR and TE10 PDR cells were cultured in medium containing 1μM palbociclib; the authentication of these cells was performed by flow cytometry analysis upon exposing to 1μM palbociclib for 24 hours (PDR cells can go through G1 arrest even in the presence of palbociclib).
Mycoplasma contamination	All cell lines were tested negative for mycoplasma contamination.
Commonly misidentified lines (See ICLAC register)	TE7 cells were used in this study. TE7 cell, harboring cyclin D1 P287A mutation, is a good model to investigate the role of cyclin D1 in regulating Gln-addiction. Morphological observation characterizes that TE7 cells can form SCC xenografts.

Animals and other organisms

Policy information about studies involving animals; ARRIVE guidelines recommended for reporting animal research

Laboratory animals	Mice species: 1) C57BL/6, Fbxo4 +/+ and −/− mice, male+female, 6 weeks old; 2) nu/nu, athymic nude with the nude spontaneous mutation (Foxn1nu, formerly Hfh11nu), male, 4 weeks old.
Wild animals	The study did not involve wild animals.
Field-collected samples	The study did not involve samples collected from the field.
Ethics oversight	The protocol was approved by the Institutional Animal Care and Use Committee (IACUC) at the Medical University of South Carolina (MUSC). The MUSC ARC # is 3339.

Note that full information on the approval of the study protocol must also be provided in the manuscript.

Flow Cytometry

Plots

Confirm that:

- [x] The axis labels state the marker and fluorochrome used (e.g. CD4-FITC).
- [x] The axis scales are clearly visible. Include numbers along axes only for bottom left plot of group (a 'group' is an analysis of identical markers).
- [ ] All plots are contour plots with outliers or pseudocolor plots.
- [x] A numerical value for number of cells or percentage (with statistics) is provided.

Methodology

Sample preparation	The flow cytometry was performed on cell lines, and the biological sources of all the cells used are indicated above.<br>The procedure for Annexin V staining: cells were firstly exposed to Gln-depletion, or relative treatment, for example, vehicle, CB, Met, CB+Met. After indicated treating time, cells will be trypsinized and suspended in medium collected before trypsinization. Cells will be washed in 1x PBS and stained by Annexin V following manufacture's protocol (BD Biosciences, Catalog No. 556420).<br>The procedure for cell cycle analysis: cells were firstly exposed to medium with or without 1μM palbociclib for 24 hours. The next day, cells were trypsinized, washed, fixed and stained with 10 μg/mL propidium iodide (PI) containing 100 μg/mL RNaseA before FACS analysis.
Instrument	BD FACSVerse™ flow cytometer
Software	The data were collected using BD FACSuite software, and the data were analyzed using FlowJo vX.0.7.
Cell population abundance	For Annexin V staining, the cell population abundance was about 80-94%; the cells were gated based on the control groups in order to keep consistency for a specific cell line.

For cell cycle analysis, the cell population abundance was about 75-80%; the cells were gated for single cell based on SSC+FSC in asynchronized group.

Gating strategy

For apoptosis analysis, FSC vs SSC plots were firstly used to gate cells and to identify any changes in the scatter properties of the cells. To define Annexin V +/− population boundaries, control and staurosporine-treated cells were utilized. The boundary for +/− was determined by setting the gate in which no cell detected for unstained cells while positive cells were shown in staurosporine-treated groups. Thereafter, apply this gating for all the other groups in one specific analysis.
For cell cycle analysis, the debris was excluded according to SSC+FSC gating and narrowed down to the single cell population. Thereafter, the DNA profile was optimized to adjust the G0/G1 peak to appear around channel 50 by changing the voltage.

☒ Tick this box to confirm that a figure exemplifying the gating strategy is provided in the Supplementary Information.
